# ADAP-METTL3 modulates the inflammatory responses of macrophages via m^6^A modification of *Spry1*

**DOI:** 10.1038/s41419-025-08008-x

**Published:** 2025-10-07

**Authors:** Anqi Dong, Pengchao Zhang, Fan Yang, Yanqi Wang, Hebin Liu

**Affiliations:** Institutes of Biology and Medical Sciences, Soochow University, Suzhou, 215123 China

**Keywords:** Methylation, Inflammation

## Abstract

While *N*^*6*^-methyladenosine (m^6^A) RNA modification is implicated in macrophage inflammatory responses, its regulatory mechanisms remain elusive. Our prior studies demonstrated that a deficiency in the immune adaptor protein ADAP promotes inflammation in TLR4-stimulated macrophages. Here, we show that ADAP binds to METTL3, and depletion of *Mettl3* alleviates the hyperinflammation in *Adap*^*−/−*^ macrophages, indicating METTL3 counteracts the anti-inflammatory function of ADAP in macrophages. Furthermore, LPS induces the METTL3-dependent m^6^A methylation of *Spry1* mRNA in macrophages in the A^6988^ within the motif GGACU, which is further potentiated upon the depletion of ADAP. Moreover, this positive effect of ADAP deficiency on LPS-induced m^6^A methylation of *Spry1* mRNA is dependent on IGF2BP2, which specifically binds to and stabilize the m^6^A modified *Spry1* mRNA that contributes to an exacerbation of the inflammation in *Adap*^*−/−*^ macrophages via NF-κB activation in macrophages. Together, our findings unveil a reciprocal inhibition between ADAP and METTL3 that fine-tunes the inflammatory responses of macrophages via modulation of the m^6^A methylation of *Spry1* mRNA.

## Introduction

The macrophage, a versatile immune cell present in most tissues, plays a central role in orchestrating inflammatory responses in tissues and contributes significantly to the development and pathogenesis of inflammation-associated diseases [1–3]. In response to microenvironmental cues, macrophages undergo dynamic switches in phenotype and function to be polarized into classically activated (M1) or alternatively activated (M2) macrophages. M1 macrophages are pro-inflammatory and play a critical role in host defense, while M2 macrophages are associated with anti-inflammatory responses and tissue repair [1, 4].

*N*^6^-methyladenosine (m^6^A) modification, one of the most prevalent internal post-transcriptional modifications of mRNA, is a dynamic and reversible process regulated by m^6^A methyltransferases (“writers”), m^6^A demethylases (“erasers”) and m^6^A-binding proteins (“readers”) [5–8]. The m^6^A methyltransferases complex, mainly including an enzymatic subunit methyltransferase-like 3 (METTL3), METTL14 and Wilms tumor 1-associating protein (WTAP), deposits m^6^A modifications in the conserved RNA consensus motif RRACH (R = G/A; H = U, A/C) [9–11]. Conversely, m^6^A demethylases, such as fat mass and obesity-associated protein (FTO) and AlkB Homolog 5 (ALKBH5), remove m^6^A modifications, ensuring the dynamic regulation of this epigenetic mark [12, 13]. The functional consequences of m^6^A modification are mediated by m^6^A reader proteins, including YT521-B homology domain-containing proteins (YTHDF1/2/3 and YTHDC1/2), insulin-like growth factor 2 mRNA-binding proteins (IGF2BP1/2/3) and heterogeneous nuclear ribonucleoprotein (HNRNP). These readers selectively recognize and bind to the m^6^A-modified transcripts, regulating mRNA stability, translation efficiency, and other post-transcriptional processes [14].

m^6^A modification plays a crucial role in regulating macrophage-mediated inflammation [15]. *Mettl3-*deficient macrophages showed reduced TNF-α production upon LPS stimulation in vitro. Furthermore, *Mettl3*^*flox/flox*^*Lyz2-Cre*^*+/−*^ mice exhibited heightened susceptibility to bacterial infections [16]. Consistently, myeloid cell-specific METTL3 deletion negatively regulated the inflammatory response and attenuated the progression in atherosclerosis [17]. These studies collectively indicated that METTL3 acts as a positive regulator of macrophage activation. However, there are inconsistent reports about the function of METTL3. It has also been reported that METTL3 knockdown significantly promoted the production of pro-inflammatory cytokines, such as TNF-α, IL-6, and nitric oxide (NO) [18]. Together, these findings underscore the complex role of m^6^A regulatory factors in regulating macrophage polarization and function. Despite these, the specific target gene of METTL3-mediated m^6^A modification in the inflammatory responses in macrophages remains poorly understood.

Adhesion and degranulation-protein adapter protein (ADAP, also known as FYB), is a hematopoietic cell-specific immune adapter protein encoded by the *Fyb* gene [19]. In macrophages, ADAP participates in both inside-out and outside-in integrin signaling and actin remodeling through the ADAP/SKAP2/Sirpα complex [20]. Our prior studies demonstrated that ADAP-deficient mice exhibit enhanced M1 polarization and heightened expression of pro-inflammatory cytokines in macrophages. Moreover, ADAP regulates the polarization and phagocytic ability of macrophages via interacting with STAT family members, specifically STAT3 and STAT1 [21, 22]. This prompted us to investigate whether m^6^A modification may contribute to the hyper-inflammatory phenotype observed in ADAP-deficient macrophages. In this study, we demonstrate that myeloid cell-specific *Mettl3* knockout ameliorates the hyper-inflammatory response of macrophages caused by ADAP deficiency in vitro and in vivo. Mechanically, we show that ADAP interacts with METTL3, and its deficiency potentiates the LPS-induced m^6^A methylation in the coding sequences (CDS)_4299_–_8747_ of *Spry1* transcripts. This m^6^A modification stabilizes *Spry1* mRNA in an IGF2BP2-dependent manner in macrophages, leading to an exacerbation of the inflammation in ADAP-deficient macrophages by activating NF-κB pathway. Our findings uncover a novel epigenetic mechanism underlying the regulatory role of ADAP in macrophage inflammatory responses.

## Results

### ADAP interacts with METTL3 in the cytoplasm of macrophages

To explore the potential link between ADAP and m^6^A methylation during inflammatory response in macrophages, the expression levels of key m^6^A regulatory components in PMs were analyzed. Specifically, the levels of m^6^A writers (METTL14, METTL3, WTAP, VIRMA, RBM15, and ELF3), erasers (FTO and ALKBH5), and readers (HNRNPC, YTHDF1/2/3, and IGF2BP1/2/3) in PMs from WT and *Adap*^*−/−*^ mice were examined after LPS stimulation or mock treatment. RT-qPCR results demonstrated that ADAP deficiency did not significantly alter the mRNA levels of these m^6^A regulators, regardless of LPS stimulation (Fig. S1A–O). In addition, there were no remarkable changes in the protein levels of the core m^6^A methyltransferase complex (METTL3, METTL14, and WTAP) or the main demethylase (FTO and ALKBH5) between WT and *Adap*^*−/−*^ PMs, with or without LPS treatment (Fig. S1P).

**Fig. 1:**
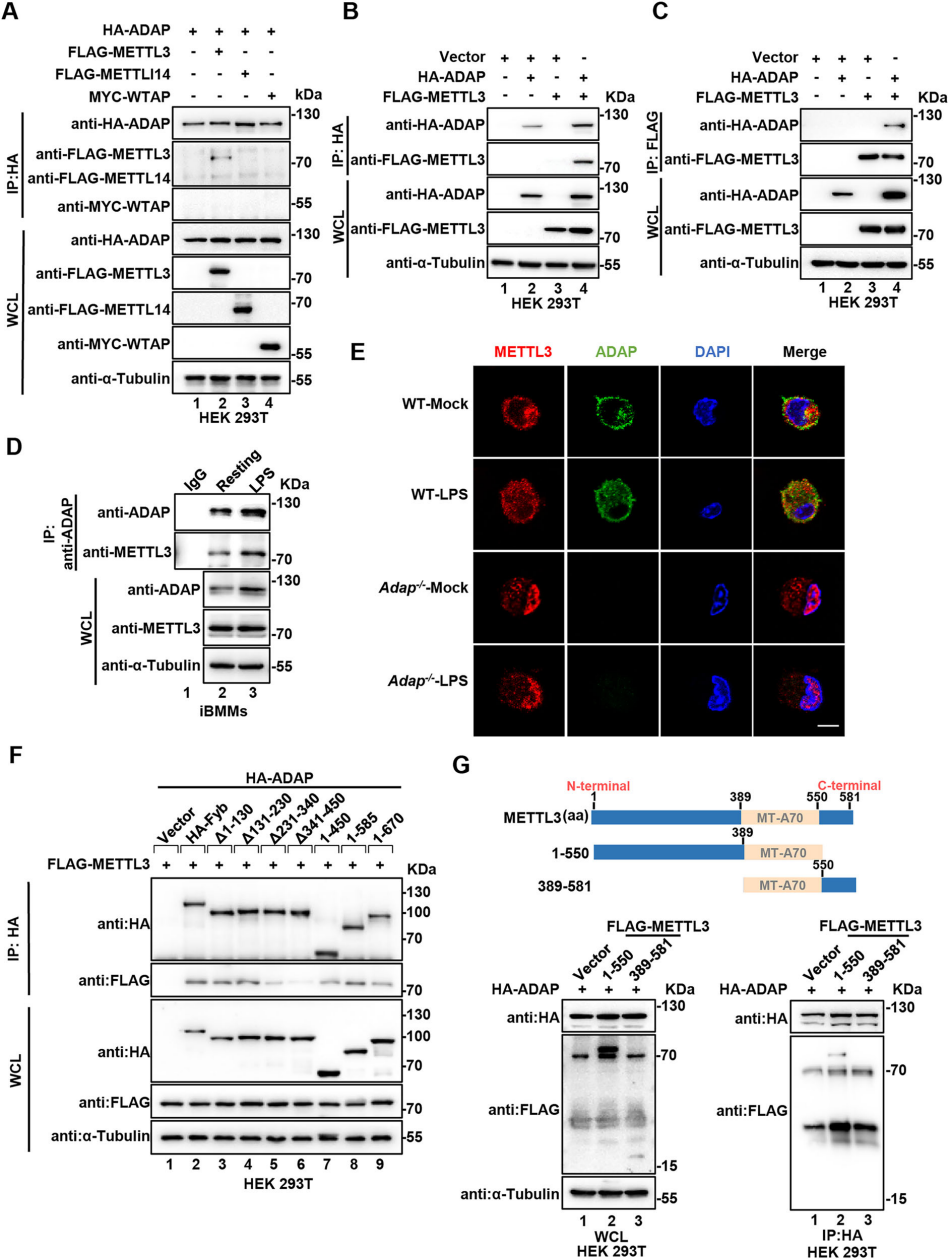
ADAP interacts with METTL3 but not METTL14 or WTAP in the cytoplasm. **A** HEK 293T cells were co-transfected with HA-ADAP, FLAG-METTL3, FLAG-METTL14, MYC-WTAP, or empty vector constructs. Forty hours post transfection, cell lysates were subjected to immunoprecipitation with an anti-HA antibody, followed by immunoblotting with the indicated antibodies. **B**, **C** HEK 293T cells were co-transfected with empty vector, FLAG-METTL3, or HA-ADAP plasmids for 36 h. Cell lysates were immunoprecipitated using either an anti-HA antibody (**B**) or an anti-FLAG antibody (**C**), followed by immunoblotting with the indicated antibodies. Whole-cell lysates were analyzed to confirm transfection efficiency. **D** iBMMs were either left untreated or stimulated with LPS (1 μg/ml). Twelve hours post stimulation, cells were lysed and subjected to immunoprecipitation (IP) using anti-ADAP or control IgG antibodies. Total and immunoprecipitated proteins were then analyzed by immunoblotting with the specified antibodies, as indicated. **E** Peritoneal macrophages (PMs) from both WT and *Adap−/*− mice were untreated or stimulated with LPS (1 μg/ml, 12 h), followed by immunofluorescence staining. Representative confocal microscopy images show the subcellular distribution and co-localization of ADAP (green) and METTL3 (red), with nuclear staining by DAPI (blue). Scale bar: 10 μm. **F** HEK 293T cells were transiently transfected with the empty vector, FLAG-METTL3, full-length HA-ADAP, or HA-ADAP mutant constructs for 40 h. Whole-cell lysates were immunoprecipitated using an anti-HA antibody, followed by immunoblotting with anti-HA or anti-FLAG antibodies. Anti-α-tubulin was used as a loading control. **G** HEK 293T cells were transfected with empty vector, full-length METTL3, or its truncated mutants, together with HA-ADAP. Forty hours post-transfection, cell lysates were immunoprecipitated using an anti-HA antibody. Immunoprecipitates were separated by SDS-PAGE and immunoblotted with anti-FLAG and anti-HA antibodies. A schematic representation of full-length METTL3 and its truncated constructs is shown.

Given the role of METTL3 in the m^6^A methyltransferase complex, we investigated whether other complex subunits, such as METTL14 and WTAP, also interact with ADAP. To this end, HEK 293 T cells were co-transfected with plasmids encoding FLAG-METTL3, FLAG-METTL14, MYC-WTAP, and HA-ADAP, followed by co-immunoprecipitation (co-IP) assays using an anti-HA antibody. Intriguingly, in vitro pull-down assays revealed that ADAP specifically interacts with FLAG-METTL3 but not with FLAG-METTL14 or MYC-WTAP (Fig. 1A). This specific interaction was further confirmed by reciprocal co-IP assays using FLAG-METTL3 and HA-ADAP in HEK 293 T cells (Fig. 1B, C). To further investigate the interaction between ADAP and METTL3 during the inflammatory response in macrophages, whole-cell lysates from WT iBMMs with or without LPS stimulation were subjected to immunoprecipitation by an anti-ADAP antibody, followed by immunoblotting with METTL3 antibody, revealing an association between endogenous ADAP and METTL3 (Fig. 1D). Immunofluorescence imaging showed that while under resting condition, a substantial portion of ADAP colocalized with METTL3 in the cytoplasm of macrophages, upon LPS stimulation, the majority of co-localization of ADAP with METTL3 was observed near the nucleus, wherein the level of their co-localization was increased. Furthermore, ADAP deficiency promoted METTL3 nuclear translocation, which was further potentiated upon LPS treatment (Fig. 1E).

To delineate the specific region of ADAP responsible for its interaction with METTL3, we generated a series of ADAP truncation mutants and co-expressed them with FLAG-tagged METTL3 in HEK 293T cells for co-immunoprecipitation assays. Our results demonstrated that the amino acid region 341–450 of ADAP is essential for its physical interaction with METTL3 in vitro (Fig. 1F). Furthermore, domain mapping of METTL3 revealed that its N-terminal region (residues 1–389), but not the C-terminal region (residues 551–581), is indispensable for ADAP binding (Fig. 1G). Collectively, these findings provide evidence for a direct physical interaction between ADAP and METTL3 in macrophages, establishing a molecular basis for their functional interplay in regulating inflammatory responses.

### The loss of METTL3 ameliorates the ADAP deficiency-induced hyperinflammatory response of macrophages to LPS stimulation

**Fig. 2:**
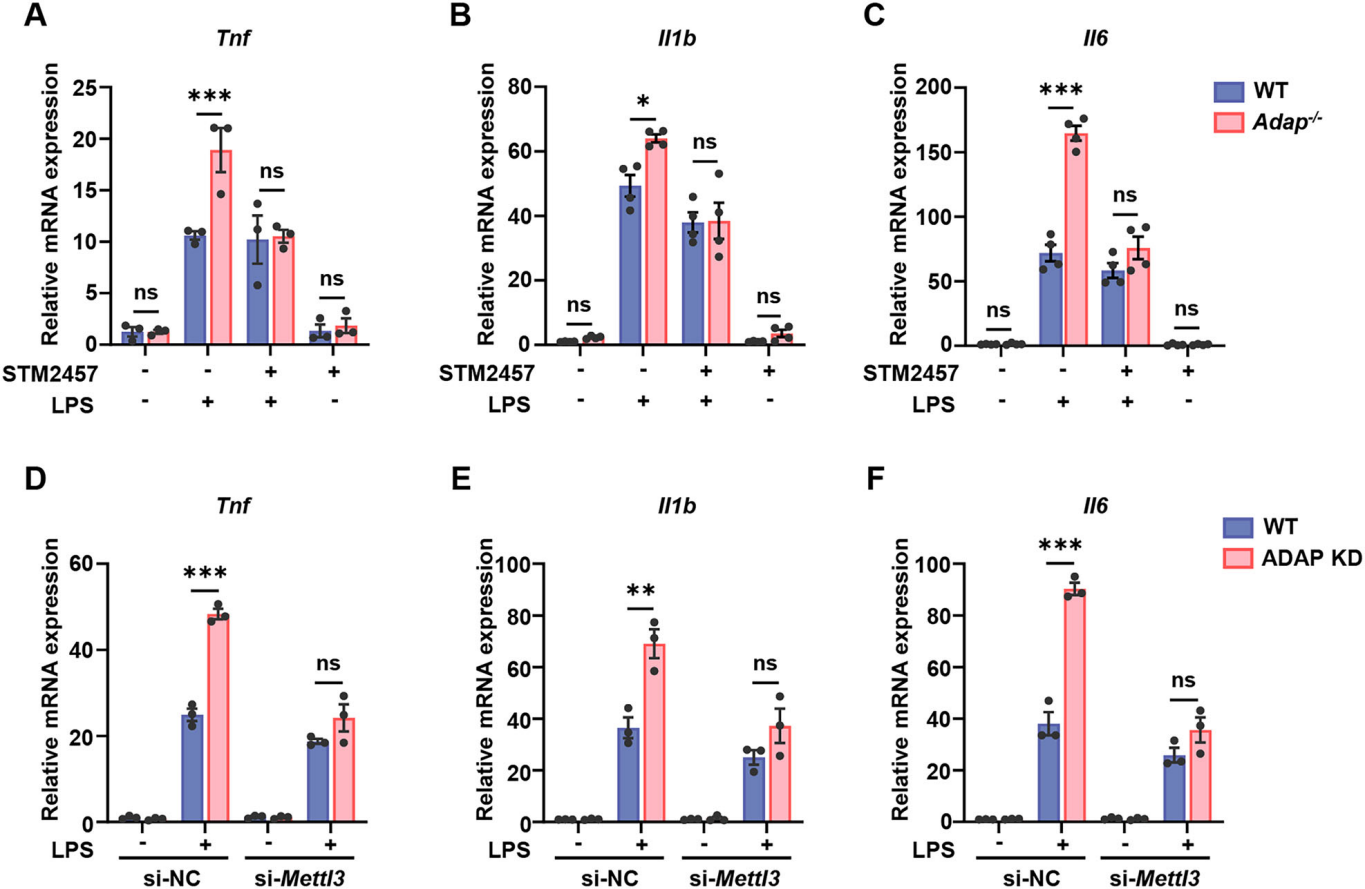
METTL3 counteracts the aggravation of inflammation caused by ADAP deficiency in TLR4-stimulated macrophages. **A**–**C** PMs isolated from WT and *Adap*^*-/-*^ mice were either untreated or treated with the METTL3-specific inhibitor STM2457 (50 μM) for 24 h. Following treatment, cells were either left unstimulated or stimulated with LPS (1 μg/ml) for 6 h. Total RNA was extracted and subjected to RT-qPCR analysis. The mRNA expression levels of TNF-α (A, *n* = 3), IL-1β (B, *n* = 4), and IL-6 (C, *n* = 4) were normalized to *Gapdh* mRNA expression. **D**–**F** WT and ADAP knockdown RAW 264.7 cells were transfected with either control siRNA (si-NC) or METTL3-specific siRNA (si-METTL3) for 24 h, followed by treatment with or without LPS (1 μg/ml, 6 h). Total RNA was extracted, and RT-qPCR analysis was performed to assess the mRNA expression levels of TNF-α (**D**), IL-1β (**E**), and IL-6 (**F**). Relative mRNA levels were normalized to *Gapdh* mRNA expression. All RT-qPCR data are presented as mean ± SEM. *n* = 3. *P* values were determined by a two-way ANOVA with multiple comparison. *P < 0.05, **P < 0.01, and ***P < 0.001.

To investigate whether METTL3-mediated m^6^A methylation contributes to the exacerbated inflammatory response in ADAP-deficient macrophages, peritoneal macrophages (PMs) isolated from WT or *Adap*^*−/−*^ mice were either stimulated with LPS or left unstimulated, followed by treatment with STM2457, a specific inhibitor of METTL3 [23]. RT-qPCR analysis revealed that LPS-induced mRNA expression of pro-inflammatory cytokines, including IL-1β, TNF-α, and IL-6, was significantly elevated in ADAP-deficient PMs compared to WT controls, consistent with our previous findings [22]. Strikingly, METTL3 inhibition markedly attenuated the hyper-inflammatory response in ADAP-deficient PMs, as evidenced by reduced cytokine levels (Fig. 2A–C), indicating that the LPS-induced hyper-inflammatory response of macrophages caused by ADAP deficiency is METTL3-dependent. To further validate these findings, we knocked down METTL3 using siRNA in RAW 264.7 cells (Fig. S2A, B). METTL3 knockdown similarly suppressed the LPS-induced upregulation of IL-1β, TNF-α, and IL-6 in ADAP-deficient macrophages (Fig. 2D–F). Together, these results demonstrate that the loss of METTL3 counteracts the potentiating effect of ADAP deficiency on the induction of pro-inflammatory cytokines in macrophages in response to TLR4 stimulation, highlighting a critical role for METTL3-mediated m^6^A methylation in regulating macrophage inflammatory responses.

### METTL3 depletion alleviates the ADAP deficiency-induced inflammatory damages via attenuation of M1 macrophage polarization in mice

To further elucidate the reciprocal relationship between METTL3 and ADAP in macrophage-mediated inflammatory responses in vivo, we generated myeloid-specific *Mettl3* conditional knockout (*cKO*) mice by crossing *Mettl3*^*fl/fl*^ mice with *Lyz2-Cre* mice, effectively abolishing *Mettl3* expression in the myeloid lineage, including macrophages (Fig. S3A, B). Compared to *Mettl3*^*fl/fl*^ (WT) mice, *cKO* mice (*Mettl3*^*fl/fl*^*Lyz2-Cre*^*+/−*^) exhibited significantly reduced METTL3 mRNA and protein levels in PMs and BMDMs, accompanied by decreased global m^6^A levels (Fig. S3C–H). To investigate the combined effects of METTL3 and ADAP deficiency, we generated double knockout (*dKO*) mice (*Mettl3*^*fl/fl*^*Lyz2-Cre*^*+/−*^*Adap*^*−/−*^), which showed marked reductions in both METTL3 and ADAP protein levels in PMs (Fig. S3I, J).

**Fig. 3:**
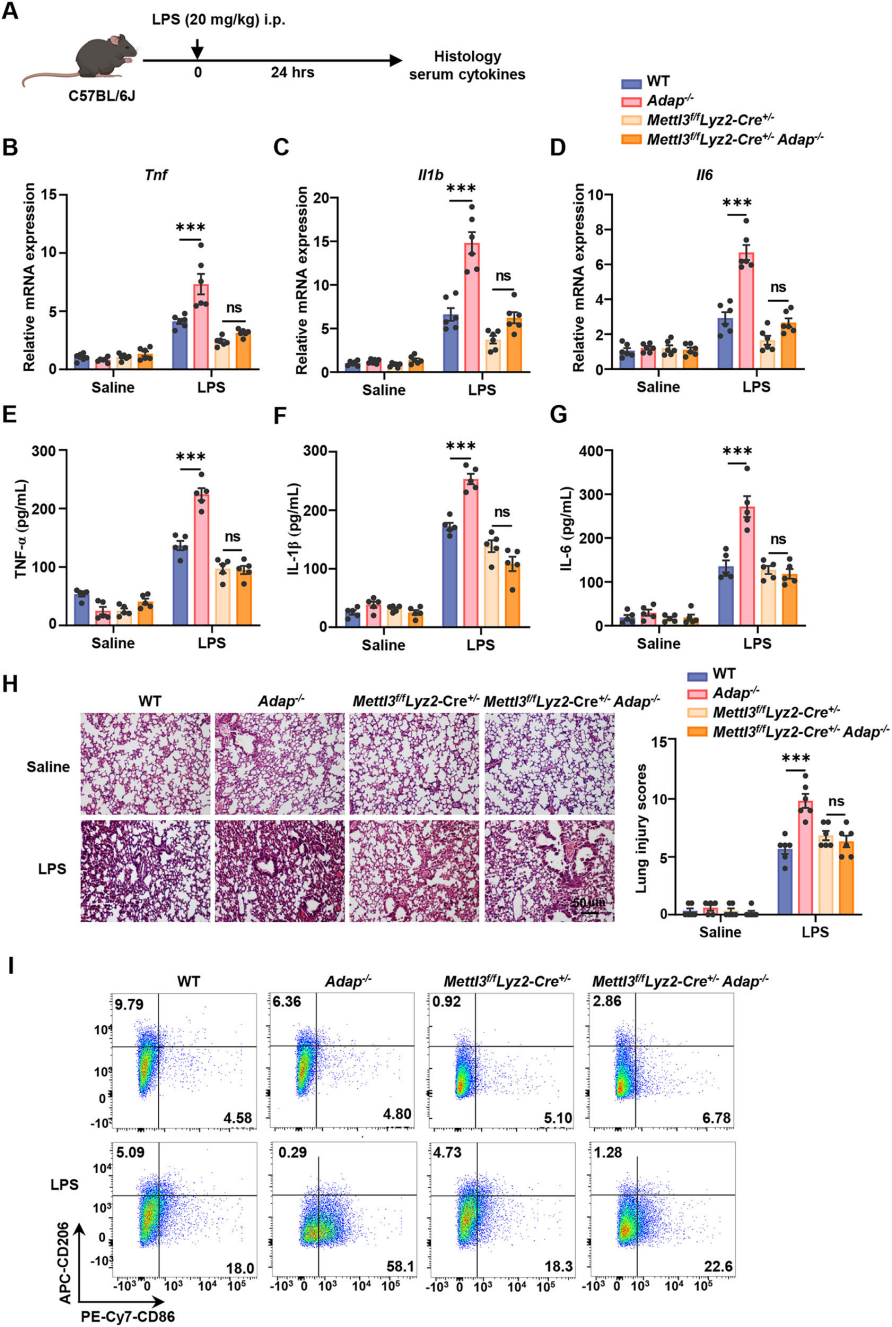
METTL3 depletion ameliorates ADAP deficiency-induced inflammatory damage in mice. **A** Experimental schema for the LPS-induced inflammatory septic mouse model. WT, *Adap*^*−/−*^, *Mettl3*^*f/f*^*Lyz2-Cre*^*+/−*^, and *Mettl3*^*f/f*^*Lyz2-Cre*^*+/−*^*Adap*^*−/−*^ mice were intraperitoneally injected with saline or LPS (20 mg/kg). Twenty-four hours post-injection, lung tissues and serum were collected. Inflammation levels in lung tissues and serum were assayed using RT-qPCR, HE staining, and ELISA. **B**–**D** WT, *Adap*^*−/−*^, *Mettl3*^*f/f*^*Lyz2-Cre*^*+/−*^, and *Mettl3*^*f/f*^*Lyz2-Cre*^*+/−*^*Adap*^*−/−*^ mice were injected with saline or LPS (20 mg/kg) for 24 h. Total RNA was extracted from lung tissues, and RT-qPCR analysis was performed to determine the relative mRNA expression of pro-inflammatory cytokines TNF-α (**B**), IL-1β (**C**), and IL-6 (**D**) (*n* = 6/group). *Gapdh* mRNA was used as an internal control to normalize the gene expression. ***P < 0.001. ns nonsignificant, two-way ANOVA. **E**–**G** WT, *Adap*^*−/*^^−^, *Mettl3*^*f/f*^*Lyz2-Cre*^*+/−*^, and *Mettl3*^*f/f*^*Lyz2-Cre*^*+/−*^*Adap*^*−/−*^ mice were intraperitoneally injected with saline or LPS for 24 h. The levels of TNF-α (**E**), IL-1β (**F**), and IL-6 (**G**) in serum were quantified by ELISA (*n* = 5/group). Data were expressed as mean ± SEM. Statistical significance was determined using two-way ANOVA. ***P < 0.001. ns nonsignificant. **H** Lung tissues from WT, *Adap*^*−/−*^, *Mettl3*^*f/f*^*Lyz2-Cre*^*+/−*^, and *Mettl3*^*f/f*^*Lyz2-Cre*^*+/−*^*Adap*^*−/*^^−^ mice were collected 24 h after intraperitoneal injection of saline or LPS (20 mg/kg) and subjected to HE staining. Scale bars: 50 μm. Quantitative assessment by lung histopathological score was presented (*n* = 6) (right panel). **I** BMDMs were isolated from WT, *Adap*^*−/−*^, *Mettl3*^*f/f*^*Lyz2-Cre*^*+/−*^, and *Mettl3*^*f/f*^*Lyz2-Cre*^*+/−*^*Adap*^*−/−*^ mice, cultured for 7 days in L929-conditioned medium, and treated with or without LPS for 12 h. Cells were stained with CD11b, F4/80, CD86, and CD206. The percentage of CD86^+^CD206^−^ cells (M1 macrophages) is shown in the lower right, and CD86^−^CD206^+^ cells (M2 macrophages) are indicated in the upper left quadrants.

To generate the mouse sepsis inflammation model induced by LPS treatment [24], WT, *Mettl3*^*fl/fl*^*Adap*^*−/−*^ (AKO), *cKO*, and *dKO* mice were intraperitoneally injected with either saline or LPS. After 24 h treatment, inflammatory damage in the lungs, liver, kidney, and serum was evaluated (Fig. 3A). RT-qPCR analysis revealed that LPS-induced mRNA levels of pro-inflammatory cytokines, including TNF-α, IL-1β, and IL-6, were significantly elevated in lung tissues of *Adap*^*−/−*^ mice compared to WT mice. Strikingly, myeloid-specific *Mettl3* deletion attenuated this hyper-inflammatory response (Fig. 3B–D). Consistently, ELISA results showed that serum levels of TNF-α, IL-1β, and IL-6 were significantly higher in *Adap*^*−/−*^ mice than in WT mice following LPS stimulation, whereas *dKO* mice exhibited reduced cytokine levels compared to AKO mice (Fig. 3E–G). In addition, survival analysis revealed that compared with the WT group, the mortality rate in the ADAP deficiency group significantly increased, whereas the METTL3-deficient group showed a markedly reduced mortality rate (Fig. S4A). Histopathological analysis of lung, liver, and kidney tissues via H&E staining revealed more severe LPS-induced tissue injury in ADAP-deficient mice compared to WT, which was ameliorated by *Mettl3* deletion in *dKO* mice (Fig. 3H and Fig. S4B–E). Furthermore, we also used the model of cecal ligation and puncture (CLP), a classic systemic inflammation model that activates the TLR4 receptor [25], to evaluate the function of the ADAP and METTL3. As shown in Supplemental Fig. 5A–F, the CLP mice exhibited significantly increased pro-inflammatory cytokine levels compared with the sham surgery group, which were further exacerbated by ADAP deficiency (bar 5 vs. bar 6). Notably, METTL3 knockout significantly suppressed the expression of pro-inflammatory cytokines in CLP mice. Histopathological analysis also revealed that the degree of tissue damage in the lung, liver, and kidney aligned with and supported conclusions drawn from the findings in the LPS-induced sepsis model (Fig. S5G–I).

Given the critical role of macrophage polarization in acute inflammation [2], we further assessed the proportions of M1 (CD86^+^) and M2 (CD206^+^) macrophage by flow cytometry. LPS stimulation increased the proportion of CD206^−^CD86^+^ M1-like macrophages in WT BMDMs, an effect exacerbated by ADAP deficiency. Notably, *Mettl3* deletion reduced the percentage of CD206^-^CD86^+^ BMDMs in *dKO* mice compared to *Adap*^*−/−*^ mice upon LPS treatment (Fig. 3I and Fig. S4D). Together, these findings demonstrate that METTL3 deficiency mitigates LPS- and CLP-induced pro-inflammatory cytokines, tissue damage, and M1 macrophage polarization in ADAP KO mice.

### *Spry1* is a target regulated by METTL3-ADAP during the inflammatory response in macrophages

**Fig. 4:**
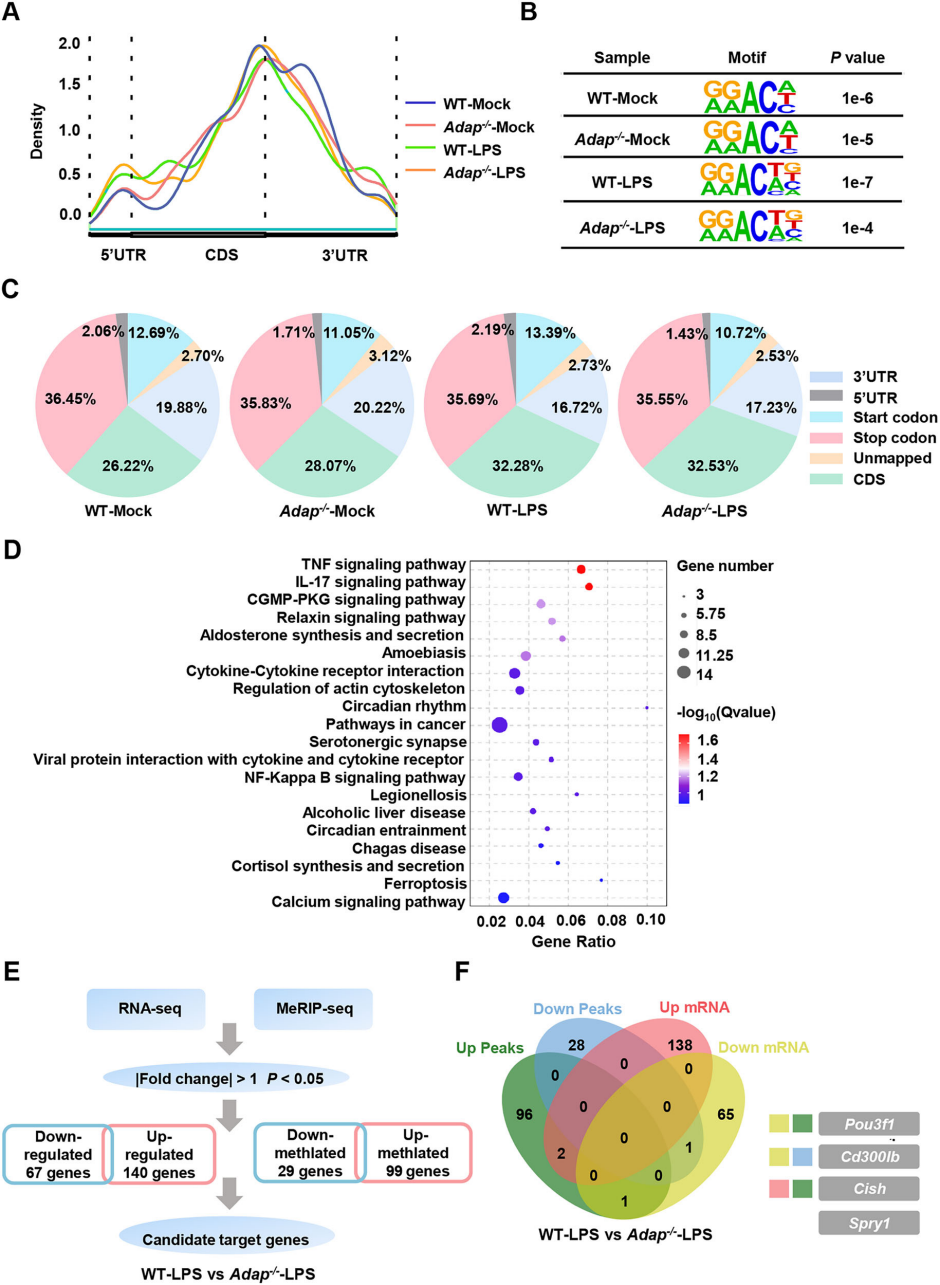
Transcriptome-wide m^6^A-seq and RNA-seq assays identify *Spry1* as a key target regulated by METTL3-ADAP in the inflammatory response of macrophages. **A** PMs derived from WT and *Adap*^*−/−*^ mice were treated with LPS (1 μg/ml) for 6 h. Total mRNA was extracted and subjected to MeRIP-seq. The density of m^6^A modification peaks across all mRNA transcripts is depicted in the graph. **B** mRNA was extracted from mock- or LPS-stimulated (1 μg/ml, 6 h) PMs and subjected to MeRIP-seq. The predominant consensus motif “GGAC” within m^6^A modification peak regions was identified using HOMER. **C** Pie charts illustrating the distribution of m^6^A peak across the 5’UTR, start codon, CDS, stop codon, and 3’UTR regions of mRNA transcripts. This analysis is based on MeRIP-seq data from PMs derived from WT and *Adap*^−/−^ mice with or without LPS (1 μg/ml, 6 h) treatment. **D** PMs from WT and *Adap*^−/^^−^ mice were either unstimulated or stimulated with LPS (1 μg/ml, 6 h). Total RNA was extracted and subjected to RNA-seq. The top 20 KEGG pathways identified from KEGG enrichment analysis of DEGs are shown. **E** A Flow chart outlining the selection process for candidate target genes of m^6^A methylation in LPS-treated PMs from WT and *Adap*^*−/−*^ mice. Differentially expressed genes (DEGs) were screened based on |fold change|>1 and *P* values <0.05. **F** Venn diagram displaying the overlap between genes with significantly altered (upregulated and downregulated) m^6^A levels in MeRIP-seq and DEGs in RNA-seq of LPS-treated PMs from WT and *Adap*^*−/−*^ mice. The overlapping genes are highlighted in the diagram.

To identify potential mRNA targets regulated by module ADAP-METTL3 in the inflammatory response of macrophages, RNA sequencing (RNA-seq) and m^6^A-methylated RNA immunoprecipitation sequencing (MeRIP-seq) were performed in LPS-stimulated PMs from WT and *Adap*^*−/−*^ mice. MeRIP-seq analysis revealed that m^6^A modifications were predominantly enriched in the 3’-UTR and internal exons (CDS), particularly near stop codons, which are common RNA sites for m^6^A modification, as reported in the previous studies (Fig. 4A, C) [9, 26]. Additionally, the most common m^6^A core motif “GGAC” was significantly enriched within m^6^A peaks in both mock-treated and LPS-treated PMs from WT and *Adap*^*−/−*^ mice (Fig. 4B). Furthermore, MeRIP-seq analysis identified 2963 and 4264 m^6^A peaks in mock-treated PMs, and 6406 and 3907 m^6^A peaks in LPS-treated PMs from WT and *Adap*^*−/−*^ mice, respectively. Among these, 99 peaks exhibited significantly increased m^6^A modifications, while 29 peaks showed significantly decreased m^6^A modifications in LPS-stimulated PMs from *Adap*^*−/−*^ mice compared to WT mice (Fig. S6A). KEGG enrichment analysis highlighted that inflammation-related signaling pathways, including the TNF-α pathways and NF-κB pathways, were strongly associated with differentially m^6^A-modified transcripts (Fig. 4D). These findings suggest that the METTL3-ADAP axis regulates specific mRNA targets through m^6^A modifications, potentially modulating key inflammatory signaling pathways in macrophages.

To identify ADAP/METTL3-regulated transcripts, we hypothesized that the most biologically relevant METTL3 targets would be those exhibiting significant changes in both m^6^A methylation and expression levels. Therefore, we integrated data from significantly altered m^6^A peaks and differentially expressed genes (DEGs) to identify transcripts regulated by m^6^A modification in LPS-treated PMs from WT and *Adap*^*−/−*^ mice. Venn overlap analysis of 128 m^6^A-modified differentially expressed transcripts (99 upregulated and 29 downregulated) from the MeRIP-seq and 207 DEGs (140 upregulated and 67 downregulated) from the RNA-seq (−1 < Log_2_FC < 1, *P* < 0.05) in LPS-treated PMs from WT and *Adap*^*−/−*^ mice (Fig. 4E). Four overlapping mRNA transcripts were identified, namely *Pou3f1*, *Cish*, *Cd300lb*, and *Spry1*, which were regulated by both m^6^A modification and ADAP deficiency in the LPS-stimulated macrophages (Fig. 4F). According to the MeRIP-seq data, the m^6^A peaks of *Pou3f1*, *Cish*, and *Spry1* transcripts were remarkably increased, whereas the m^6^A peaks of *Cd300lb* were significantly decreased in *Adap*^*−/−*^ PMs compared with WT PMs (Fig. S6B–E). Consistent with the RNA-seq data, RT-qPCR validation confirmed the expression changes of these transcripts (Fig. S6F–I). Notably, *Spry1*, a negative regulator of receptor tyrosine kinase signaling, has been implicated in modulating inflammatory responses and is associated with various inflammatory diseases [27, 28]. Hence, we focused on *Spry1* as a novel target of METTL3 in macrophages and is subjected to regulation by ADAP.

### LPS induces METTL3-dependent m^6^A methylation of *Spry1* mRNA, which is negatively regulated by ADAP

**Fig. 5:**
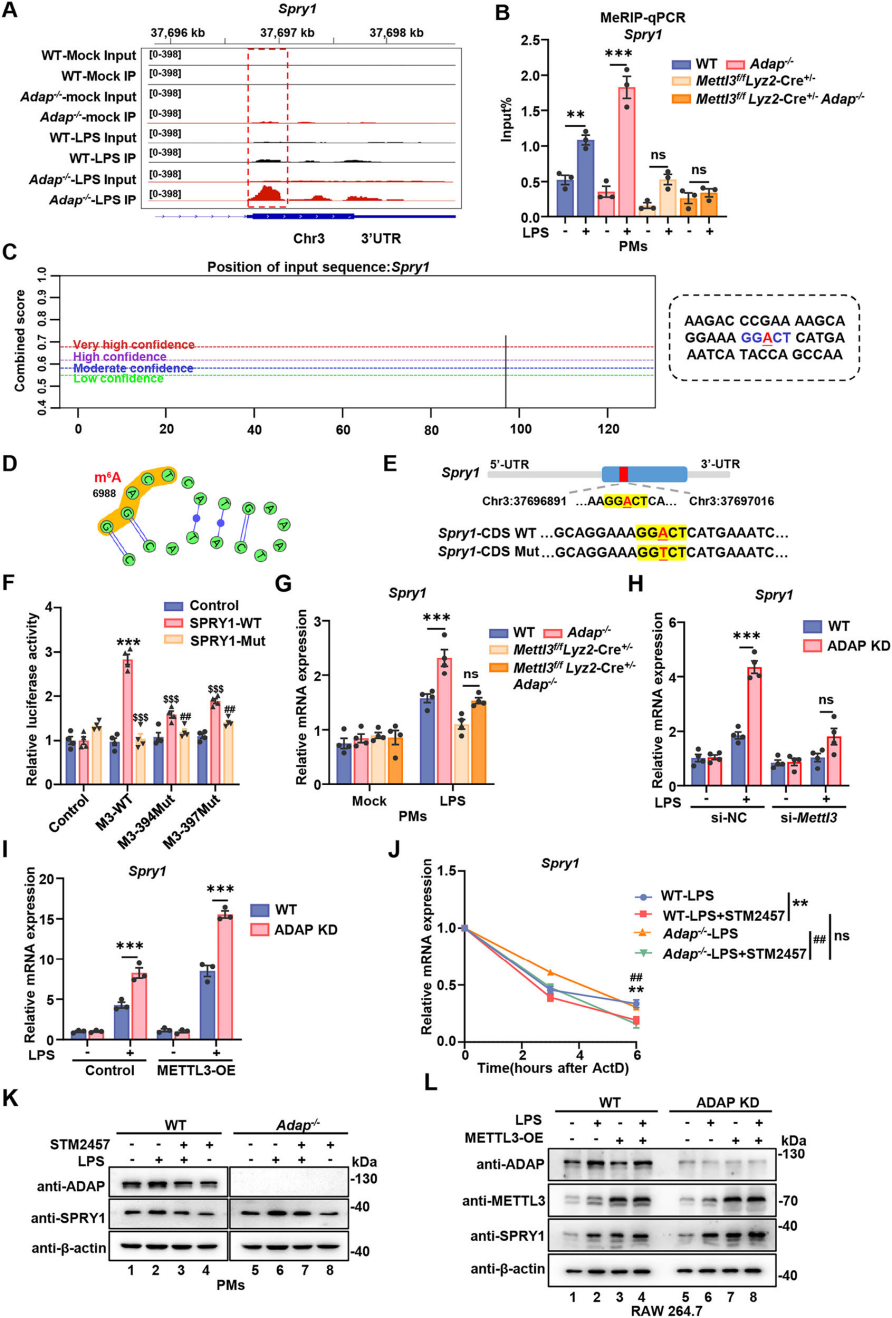
ADAP deficiency enhances the METTL3-mediated m^6^A methylation level of *Spry1* mRNA in macrophages in response to LPS stimulation. **A** PMs from WT and *Adap*^*−/−*^ mice were either unstimulated or stimulated with LPS (1 μg/ml, 6 h). Isolated RNA was subjected to MeRIP-seq. Integrative Genomics Viewer (IGV) tracks display the distribution of m^6^A-modified peaks on *Spry1* transcripts. **B** PMs derived from WT, *Adap*^*−/−*^, *Mettl3*^*f/f*^*Lyz2-Cre*^*+/−*^, and *Mettl3*^*f/f*^*Lyz2-Cre*^*+/−*^*Adap*^*−/−*^ mice were treated with or without LPS (1 μg/ml, 6 h). Total RNA was extracted, fragmented, and immunoprecipitated using anti-m^6^A or IgG antibodies. The relative quantity of *Spry1* mRNA immunoprecipitated by the m^6^A antibody compared to IgG was assayed by RT-qPCR. *n* = 3. **P < 0.01, ***P < 0.001. ns nonsignificant. **C**, **D** The putative m^6^A site in the *Spry1* mRNA transcript was predicted using the SRAMP online tool (http://www.cuilab.cn/). **E** A schematic representation of wild-type or mutant (A to T mutation) m^6^A sites in the *Spry1*-CDS (6891–7016), which was cloned into a dual-luciferase reporter vector. **F** HEK 293 T cells were transiently transfected with wild-type or mutant METTL3 plasmid (D394A and W397A), alongside SPRY1 luciferase reporters containing either m^6^A sites or mutated sites, and either empty vector or pRL-Renilla luciferase plasmids. Luciferase reporter assays were conducted 48 h post-transfection. Relative firefly luciferase activity, normalized to Renilla luciferase, is presented as mean ± SEM. *n* = 4 for each group. Statistical significance was determined using two-way ANOVA followed by multiple comparisons: ***P < 0.001 compared to the control group; ^$$$^*P* < 0.001 compared to the M3-WT group; and ^##^*P* < 0.01 compared to the SPRY1-Mut group. **G** PMs from WT, *Adap*^*−/−*^, *Mettl3*^*f/f*^*Lyz2-Cre*^*+/−*^, and *Mettl3*^*f/f*^*Lyz2-Cre*^*+/−*^*Adap*^*−/−*^ mice were treated with or without LPS (1 μg/ml) for 6 h. Total RNA was extracted, and the relative expression of *Spry1* mRNA was assayed by RT-qPCR. *P < 0.05, ***P < 0.001. **H** WT and ADAP knockdown RAW 264.7 cells were transfected with non-targeting control siRNA (si-NC) or siRNA targeting METTL3 (si-METTL3) for 24 h, followed by mock or LPS (1 μg/ml) treatment for 6 h. RNA was extracted and subjected to RT-qPCR. The relative *Spry1* mRNA level was normalized to *Gapdh* mRNA. *P < 0.05, ***P < 0.001. **I** WT and ADAP knockdown RAW 264.7 cells were transduced with a lentivirus vector containing METTL3 or an empty vector control for 48–72 h. After 7–14 days of puromycin selection, cells were left unstimulated or stimulated with LPS (1 μg/ml) for 6 h. Total RNA was extracted and subjected to RT-qPCR analysis of *Spry1* mRNA. Relative mRNA levels were normalized to *Gapdh*. **J** PMs from WT and *Adap*^*−/*^^−^ mice were pre-treated with PBS or STM2457 (50 μM) for 24 h. Cells were then left unstimulated or stimulated with LPS (1 μg/ml, 6 h), followed by treatment with actinomycin D (ActD) (5 μg/ml) for the indicated times. The decay of *Spry1* mRNA was assayed by RT-qPCR (normalized to 0 h). Data were representative of three independent experiments and analyzed by two-way ANOVA with multiple comparison. **P < 0.01. ns nonsignificant. **K** PMs from WT or *Adap*^*−/−*^ mice were treated with PBS or STM2457 (50 μM) for 24 h, followed by 6 h of stimulation with PBS or LPS. ADAP and SPRY1 protein levels in cell lysates were analyzed by Western blotting using the indicated antibodies. β-actin was used as a loading control. **L** WT and ADAP knockdown RAW 264.7 cells were transduced with METTL3-overexpressing lentivirus or control lentivirus. After 48–72 h, cells were selected with puromycin for 7–14 days. Cells were treated with or without LPS (1 μg/ml) for 6 h followed by lysis and immunoblotting with indicated antibodies.

Given that *Spry1* is potentially regulated by the ADAP-METTL3 module, we performed Integrative Genomics Viewer (IGV) analysis of *Spry1* mRNA using MeRIP-seq data. In the absence of LPS stimulation, no significant m^6^A peaks were observed in *Spry1* mRNA in PMs isolated from either WT or *Adap*^*−/−*^ mice. However, upon LPS stimulation, a high density of m^6^A peaks was detected within the CDS of *Spry1* mRNA, indicating that the m^6^A modification of *Spry1* mRNA is inducible in macrophages following LPS exposure. Intriguingly, the density of these LPS-induced m^6^A modifications was markedly higher in PMs from *Adap*^*−/−*^ mice compared to those from WT mice (Fig. 5A). These findings suggest that the m^6^A peaks in *Spry1* mRNA are specifically induced by LPS, which is negatively regulated by ADAP.

To further investigate whether the positive effect of METTL3 on *Spry1* mRNA expression is dependent on m^6^A modification, MeRIP-qPCR was performed. As expected, the m^6^A levels of *Spry1* mRNA were noticeably elevated in PMs of WT mice following LPS treatment, and this induction of m^6^A modification was more pronounced in PMs from *Adap*^*−/−*^ mice, consistent with the MeRIP-seq data. Importantly, the LPS-induced enhancement in m^6^A level of *Spry1* was substantially attenuated upon *Mettl3* depletion, as assessed in PMs from both METTL3 *cKO* and METTL3-ADAP *dKO* mice (Fig. 5B). These results demonstrate that ADAP-mediated regulation of LPS-induced m^6^A modification of *Spry1* mRNA is dependent on METTL3 activity. Since the hypermethylated m^6^A peaks of *Spry1* are primarily located within the CDS, specific m^6^A sites on the CDS of *Spry1* were predicted using the online m^6^A site predictor SRAMP. One high-confidence site, A^6988^, was identified within the putative motif “GGACT” in the CDS region (6891–7016) of *Spry1* (Fig. 5C, D). To further address whether the effect of m^6^A modification on the *Spry1* mRNA relies on the site A^6988^ within this motif, luciferase reporter plasmids were constructed, containing either wild-type CDS_6891–7016_ of *Spry1* or a mutated version in which the m^6^A site was replaced with thymine (T) (Fig. 5E). In addition, we generated two METTL3 mutants, D394A and W397A, which are defective in m^6^A catalytic activity, to further dissect the role of METTL3 in this process [29]. Luciferase reporter assays revealed that the presence of wild-type METTL3, but not the catalytically inactive mutants, significantly enhanced the luciferase activity of the wild-type *Spry1*-CDS construct compared to the m^6^A-mutated version (Fig. 5F). These findings suggest that METTL3-mediated m^6^A modification at the A^6988^ site is critical for the regulation of *Spry1* mRNA. Furthermore, LPS-induced upregulation of *Spry1* mRNA expression was significantly higher in PMs from *Adap*^*−/−*^ mice compared to those from WT mice. Again, this enhanced induction of *Spry1* mRNA in *Adap*^*−/−*^ PMs was abolished upon METTL3 knockout (Fig. 5G) or upon METTL3 knockdown using small interfering RNA targeting *Mettl3* (Fig. 5H).

To further explore the interplay between METTL3 and ADAP in regulating *Spry1* expression, we overexpressed METTL3 in both WT and ADAP knockdown RAW 264.7 cells using lentiviral-mediated gene delivery (Fig. S2C, D). As expected, METTL3 overexpression resulted in a significant increase in *Spry1* mRNA levels in both WT and ADAP knockdown RAW 264.7 cells. However, in comparison, the mRNA level of *Spry1* was much higher in ADAP knockdown cells than in WT cells (Fig. 5I), suggesting that METTL3 positively regulates *Spry1* mRNA, in which ADAP is detrimental to this positive effect of METTL3.

To analyze the effect of m^6^A modification on *Spry1* mRNA stability, we performed the RNA stability assay in PMs from WT and *Adap*^*−/−*^ mice. METTL3 inhibition reduced the half-life of *Spry1* mRNA in PMs from both WT and *Adap*^*−/−*^ mice, indicating that the upregulation of *Spry1* was partly at least due to the increased stability of the *Spry1* mRNA affected by METTL3-mediated m^6^A modification (Fig. 5J). Consistent with these findings, Western blot analysis demonstrated that SPRY1 protein levels were also upregulated in LPS-treated PMs from both WT and *Adap*^*−/−*^ mice; with a more pronounced induction observed in *Adap*^*−/−*^ PMs. Importantly, this induction was robustly attenuated following METTL3 inhibition with STM2457 (Fig. 5K). Furthermore, METTL3 overexpression in RAW 264.7 cells resulted in elevated SPRY1 protein levels following LPS stimulation, with a more substantial increase observed in ADAP knockdown cells compared to WT cells (Fig. 5L). Taken together, these results suggest that METTL3 positively regulates *Spry1* mRNA in an m^6^A-dependent manner, and this process is negatively regulated by ADAP in macrophages.

### IGF2BP2 stabilizes *Spry1* mRNA in an m^6^A-dependent manner and knock-down of IGF2BP2 restricts the hyper-inflammatory responses in *Adap*^*−/−*^ macrophages

**Fig. 6:**
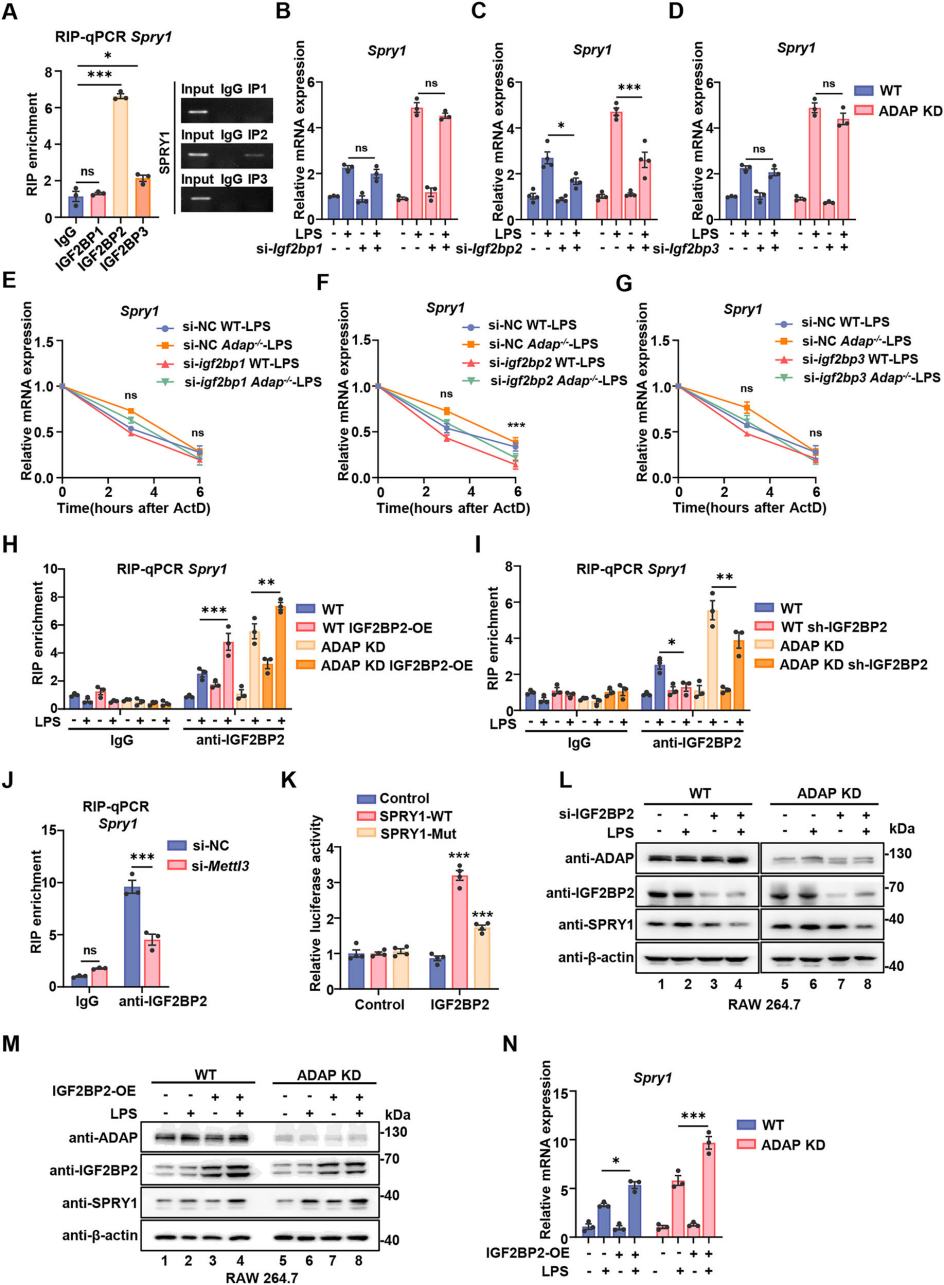
IGF2BP2 enhances the stability of methylated *Spry1* mRNA in an m^6^A-dependent manner in ADAP-deficient macrophages. **A** RAW 264.7 cells were lysed, and cell lysates were immunoprecipitated with anti-IGF2BP1, anti-IGF2BP2, or anti-IGF2BP3 antibodies, along with corresponding control IgG antibodies. Immunoprecipitated RNA was isolated and reverse-transcribed into cDNA. Relative *Spry1* mRNA enrichment was assayed by RT-qPCR (left panel) and agarose electrophoresis (right panel). Unpaired Student’s *t*-test. *n* = 3. *P < 0.05, ***P < 0.001 and ns nonsignificant. **B**–**D** WT and ADAP knockdown RAW 264.7 cells were transfected with siRNA targeting IGF2BP1 (**B**), IGF2BP2 (**C**), IGF2BP3 (**D**), or non-targeting control siRNA (si-NC) for 24 h, followed by treatment with PBS or LPS. Cells were harvested for total RNA isolation, and the expression levels of *Spry1* mRNA were assayed by RT-qPCR. Values were normalized to *Gapdh* mRNA expression for each sample. Statistical significance was determined using a two-way ANOVA with multiple comparison. *P < 0.05, **P < 0.01, ***P < 0.001 and ns nonsignificant. **E**–**G** WT and ADAP knockdown RAW 264.7 cells were transfected with control siRNA (si-NC), or siRNA targeting IGF2BP1 (**E**), IGF2BP2 (**F**), or IGF2BP3 (**G**). Twenty-four hours post-transfection, cells were treated in PBS or LPS (1 μg/ml) for 6 h, followed by treatment with ActD for the indicated times. Total RNA was extracted, and the half-life of *Spry1* mRNA was measured by RT-qPCR (normalized to 0 h). *n* = 3, two-way ANOVA with multiple comparison. **P < 0.01, ns nonsignificant. **H**, **I**, WT and ADAP knockdown RAW 264.7 cells were infected with either a lentivirus vector carrying IGF2BP2 overexpression (OE) (**H**) or a lentivirus expressing shRNA targeting IGF2BP2 (**I**) for 48 h. Following puromycin selection for 7–14 days, cells were cultured in the presence or absence of LPS for 6 h. RNA was extracted and immunoprecipitated with anti-IGF2BP2 or IgG antibodies. The enrichment of IGF2BP2 binding to *Spry1* mRNA was assayed by RT-qPCR. **J** RAW 264.7 cells transfected with either control siRNA or siRNA targeting METTL3 were collected and lysed 24 h post transfection. Cell lysates were immunoprecipitated with anti-IGF2BP2 or control IgG antibodies, followed by RNA extraction for RT-qPCR analysis. *n* = 3. Two-way ANOVA with multiple comparison was used for statistical analysis. ***P < 0.001 and ns nonsignificant. **K** HEK 293 T cells were co-transfected with an IGF2BP2 plasmid and either a wild-type or mutant SPRY1 luciferase reporter plasmid, along with an empty vector control or a Renilla luciferase reporter plasmid for 48 h. Firefly luciferase activity was measured and normalized to Renilla luciferase activity. **L** WT and ADAP knockdown RAW 264.7 cells were transfected with non-targeting control siRNA or IGF2BP2-specific siRNA for 48 h. Protein expression of ADAP, IGF2BP2 and SPRY1 were measured by Western blotting using the indicated antibodies. β-actin was used as an internal loading control. **M**, **N** WT and ADAP knockdown RAW 264.7 cells were transfected with either a lentivirus vector control or an IGF2BP2 overexpression plasmid for 48–72 h. After 7–14 days of puromycin selection, cells were left unstimulated or stimulated with LPS (1 μg/ml) for 6 h. Cells were collected and lysed, followed by immunoblotting with the indicated antibodies (**M**). Total RNA was extracted, and the relative mRNA level of *Spry1* was assayed by RT-qPCR (**N**).

Our results demonstrate that METTL3-mediated m^6^A modification of *Spry1* mRNA promotes its mRNA stability (Fig. 5). Given that members of the IGF2BP (insulin-like growth factor 2 binding protein) family are known to play a key role in stabilizing m^6^A-modified mRNAs [30], we thus assessed the potential involvement of IGF2BP1, IGF2BP2 and IGF2BP3 in the regulation of *Spry1* mRNA. RNA immunoprecipitation (RIP) assays using anti-IGF2BP1, anti-IGF2BP2, and IGF2BP3 antibodies revealed that IGF2BP2, but not IGF2BP1 or IGF2BP3, directly binds to *Spry1* mRNA in RAW 264.7 cells (Fig. 6A). Furthermore, RT-qPCR analysis demonstrated that siRNA-mediated knockdown of IGF2BP2, but not IGF2BP1 or IGF2BP3, noticeably reduced *Spry1* mRNA levels in ADAP knockdown RAW 264.7 macrophages compared to WT RAW 264.7 cells, regardless of LPS treatment (Fig. S2E–J and Fig. 6B–D). Consistent with these findings, RNA stability assays demonstrated that the half-life of *Spry1* mRNA was significantly decreased in RAW 264.7 cells transfected with IGF2BP2-specific siRNA, but not with siRNAs targeting IGF2BP1 and IGF2BP3 (Fig. 6E–G). These results suggest that IGF2BP2 specifically regulates *Spry1* mRNA stability within the METTL3-ADAP regulatory module during the inflammatory response of macrophages.

To further characterize the role of IGF2BP2 in the regulation of *Spry1*, stable cell lines of WT and ADAP knockdown RAW 264.7 with either IGF2BP2 knockdown or overexpression were constructed and verified by Western blotting (Fig. S2K–N). RIP-qPCR analysis revealed that the binding efficiency of IGF2BP2 to *Spry1* mRNA was significantly higher in ADAP knockdown RAW 264.7 cells compared to WT cells following LPS treatment, due to increased m^6^A modification levels. Overexpression of IGF2BP2 enhanced, while knockdown of IGF2BP2 decreased, the tensity of IGF2BP2 in binding *Spry1* mRNA in both WT and ADAP knockdown RAW 264.7 cells (Fig. 6H, I). To verify whether the binding of IGF2BP2 to *Spry1* mRNA depends on METTL3-mediated m^6^A methylation, RIP-qPCR assays were performed following METTL3 knockdown in RAW 264.7 cells. As expected, the binding of IGF2BP2 to *Spry1* mRNA was significantly reduced in RAW 264.7 cells transfected with METTL3-specific siRNA compared to control siRNA-transfected cells (Fig. 6J).

The binding of IGF2BP2 to the m^6^A site of *Spry1* mRNA was further determined by luciferase reporter assays. Mutation of the m^6^A site in *Spry1* mRNA significantly decreased the luciferase activity associated with IGF2BP2 binding compared to the wild-type construct (Fig. 6K), indicating that IGF2BP2 binding to *Spry1* mRNA is dependent on METTL3-mediated m^6^A methylation. Moreover, Western blot analysis demonstrated that IGF2BP2 knockdown reduced *Spry1* protein levels in both WT and ADAP knockdown RAW 264.7 cells upon LPS stimulation (Fig. 6L). Conversely, IGF2BP2 overexpression further potentiates the LPS-induced upregulation of *Spry1* mRNA and protein levels in both WT and ADAP KD RAW 264.7 cells (Fig. 6M, N). Taken together, our data demonstrate that METTL3-mediated m^6^A modification enhances *Spry1* mRNA expression by promoting IGF2BP2-dependent stabilization of *Spry1* mRNA in macrophages.

### SPRY1 sustains the hyperinflammatory response of *Adap*^*−/−*^ macrophages via the NF-κB pathway

**Fig. 7:**
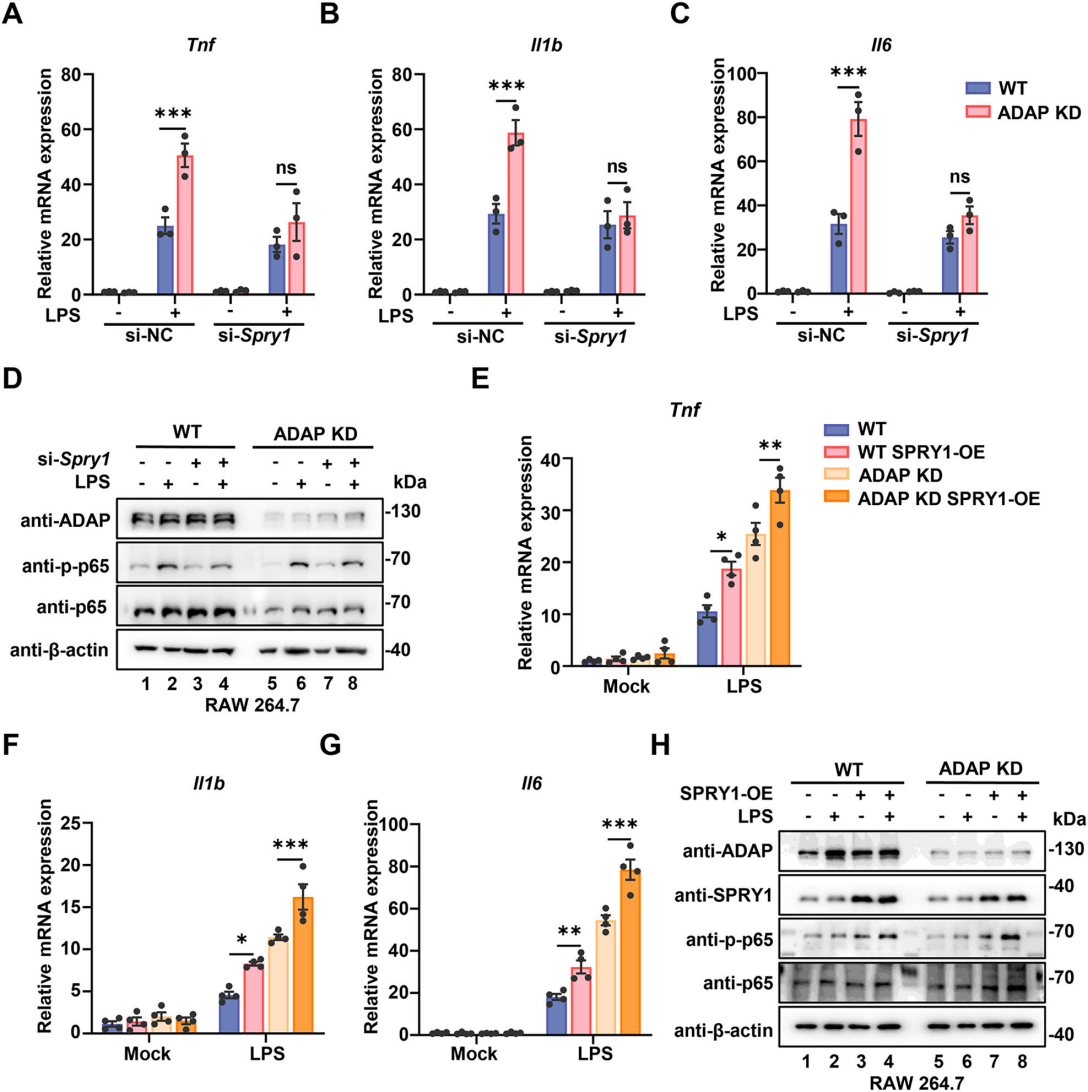
SPRY1 activates inflammation via NF-κB-dependent mechanisms in ADAP-deficient macrophages. **A**–**C** WT and ADAP knockdown RAW 264.7 cells were transfected with non-targeting control siRNA (si-NC) or siRNA targeting SPRY1 (si-SPRY1). Twenty-four hours post-transfection, cells were either left untreated or stimulated with LPS (1 μg/ml, 6 h). RT-qPCR results showing the mRNA expression of TNF-α (**A**), IL-1β (**B**), and IL-6 (**C**) are presented. *n* = 3. Data are expressed as mean ± SEM. *P* values were from a two-way ANOVA with multiple comparison. ***P < 0.001. ns nonsignificant. **D** WT and ADAP knockdown RAW 264.7 cells were transfected with either SPRY1-specific siRNA or non-targeting siRNA as a control. Forty-eight hours post transfection, cells were either left unstimulated or stimulated with 1 μg/ml LPS for 15 min. Whole-cell lysates were collected and subjected to Western blot analysis using anti-phospho-p65 and anti-p65 antibodies. **E**–**G** WT and ADAP knockdown RAW 264.7 cells were reconstituted via lentiviral transduction with either an empty vector or a SPRY1 overexpression (SPRY1-OE) vector. Cells were left unstimulated or stimulated with LPS (1 μg/ml) for 6 h. Total RNA was extracted and subjected to RT-qPCR analysis for mRNA expression of TNF (**E**), IL-1β (**F**), and IL-6 (**G**). Data were normalized to *Gapdh* expression. Statistical significance was assessed by an unpaired two-tailed Student *t*-test. *P < 0.05, **P < 0.01, ***P < 0.001. **H** WT and ADAP knockdown RAW 264.7 cells were reconstituted via lentiviral transduction with either an empty vector or a SPRY1 overexpression (SPRY1-OE) vector. Cells were left unstimulated or stimulated with LPS (1 μg/ml) for 15 min. Cell lysates were subjected to Western blot analysis using the indicated antibodies.

To investigate the functional roles of SPRY1 in LPS-induced hyperinflammation in ADAP-deficient macrophages, small interfering RNA was used to silence *Spry1* (Fig. S2O, P). Knockdown of *Spry1* ameliorated the ADAP deficiency-induced hyper-production of pro-inflammatory cytokines IL-1β, IL-6, and TNF-α in TLR4-stimulated RAW 264.7 cells (Fig. 7A–C). To further elucidate the mechanism underlying SPRY1-mediated hyperinflammation, we examined the activation of the NF-κB signaling pathway in both WT and ADAP-deficient RAW 264.7 cells following *Spry1* knockdown and LPS stimulation. Western blot analysis revealed that LPS-induced phosphorylation of p65, a key component of the NF-κB pathway, was significantly reduced in si-SPRY1-transfected RAW 264.7 cells compared to control siRNA-transfected cells. Notably, no significant differences in p65 phosphorylation levels were observed between WT and ADAP-deficient RAW 264.7 cells after *Spry1* knockdown (Fig. 7D). These findings indicate that elevated SPRY1 expression contributes to the hyperinflammatory response in ADAP-deficient macrophages by enhancing NF-κB pathway activation.

To further validate the role of SPRY1 in regulating macrophage inflammatory responses, a stable RAW 264.7 cell line overexpressing *Spry1* using lentiviral-mediated gene delivery was established (Fig. S2Q, R). Compared to WT cells, *Spry1* overexpression significantly increases the mRNA levels of pro-inflammatory cytokines IL-1β, IL-6, and TNF-α in ADAP-deficient RAW 264.7 cells, with even greater upregulation observed in the context of ADAP deficiency (Fig. 7E–G). Consistent with these findings, *Spry1* overexpression also upregulated LPS-induced phosphorylated p65 in both WT and ADAP-deficient RAW 264.7 cells, with higher levels of phosphorylated p65 observed in ADAP-deficient cells (Fig. 7H). Collectively, these results demonstrate that elevated SPRY1 expression drives hyperinflammation in ADAP-deficient macrophages by activating the NF-κB signaling pathway, leading to increased production of pro-inflammatory cytokines.

## Discussion

To this end, we demonstrate that the ADAP-METTL3 signaling axis plays a critical role in regulating the inflammatory response in macrophages via m^6^A-dependent post-transcriptional modification of *Spry1*. We showed that *Mettl3* knockout ameliorates the hyper-inflammatory response of macrophages caused by ADAP deficiency in vitro and in vivo, indicating METTL3 counteracts the ADAP effect on the inflammatory response of macrophages. Mechanically, ADAP interacts with METTL3, and ADAP deficiency potentiates LPS-induced m^6^A methylation within the CDS region of *Spry1* transcripts. This m^6^A modification is essential for the stabilization of *Spry1* mRNA, a process mediated by the m^6^A reader protein IGF2BP2. Notably, METTL3-deficient macrophages exhibit nearly complete loss of LPS-induced upregulation of *Spry1*, underscoring the critical role of METTL3 in this regulatory process. Furthermore, we reveal that SPRY1 exacerbates inflammation in ADAP-deficient macrophages by activating the NF-κB pathway. Collectively, our findings uncover a novel mechanism whereby ADAP forms a complex with METTL3 to restrain the METTL3-mediated m^6^A modification of *Spry1* destined for IGF2BP2 recognition. These findings uncover an unexpected function for the ADAP in the METTL3-dependent targeting of *Spry1* mRNA for m^6^A modification in macrophages and suggest a crucial biological role for METTL3-dependent *Spry1* mRNA m^6^A modification in inflammatory disease development.

Our previous studies demonstrated that LPS stimulation upregulates ADAP expression in macrophages, and ADAP-null macrophages display an enhanced inflammatory response and M1 polarization upon LPS stimulation [22]. We further discovered that this hyper-inflammatory response in ADAP-deficient macrophages is dependent on METTL3, as it was effectively prevented by either siRNA-mediated knockdown or pharmacological inhibition of METTL3 (Fig. 2). Additionally, myeloid-specific deletion of *Mettl3* mitigated LPS-induced severe inflammatory injury in ADAP-deficient mice in vivo (Fig. 3). Interestingly, our data also identified a physical interaction between ADAP and METTL3, suggesting a direct regulatory relationship between these two proteins. Thus, these findings collectively indicate that METTL3, a key methyltransferase, plays a pro-inflammatory role in the inflammatory response of macrophages. In contrast, ADAP appears to counteract the pro-inflammatory effects of METTL3, highlighting an opposing regulatory role in macrophage inflammation. This finding is in line with previous reports that METTL3 acts as a positive regulator of inflammation in macrophages [31, 32].

The MeRIP-seq combined with RNA-seq analysis identified *Spry1* as a target gene regulated by the METTL3-ADAP module in macrophage inflammatory responses. SPRY1, a member of the sprouty gene family, is known to function as a negative regulator of receptor tyrosine kinase (RTK) signal transduction [33, 34]. Recent studies have also implicated SPRY1 in macrophage-mediated inflammatory responses, including the activation of NF-κB signaling and the promotion of macrophage infiltration via the CXCL12-CXCR4 axis in pancreatic cancer [27]. However, the regulation of *Spry1* by m^6^A modification in macrophages has not been previously reported. In this study, we provide compelling evidence that *Spry1* mRNA undergoes m^6^A modified in macrophages, and this m^6^A modification is significantly enhanced upon LPS stimulation. Using luciferase reporter and mutagenesis assays, we further demonstrated that LPS-induced m^6^A modification predominantly occurs within the CDS region of *Spry1* mRNA. Importantly, METTL3 is responsible for increasing the m^6^A levels in the CDS region of *Spry1*, which is essential for its role in regulating inflammatory responses of macrophages. Notably, the level of LPS-induced m^6^A modification in *Spry1* transcripts was markedly higher in *Adap*^*-/-*^ macrophages compared to WT macrophages, suggesting that ADAP acts as a negative regulator of METTL3-mediated m^6^A modification of *Spry1*.

We provide compelling evidence that m^6^A methylation plays a critical role in regulating *Spry1* mRNA stability in macrophages. LPS-stimulated ADAP-deficient macrophages exhibited elevated m^6^A levels in *Spry1* transcripts, leading to a significant increase in *Spry1* expression at both the mRNA and protein levels. This enhanced expression of SPRY1 is associated with amplified macrophage inflammatory responses, mediated through the activation of the NF-κB signaling pathway. To our knowledge, this is the first time to demonstrate that the ADAP-METTL3 module has been shown to participate in the process of macrophage inflammatory responses via METTL3-mediated m^6^A methylation of *Spry1*.

Our results revealed that knockdown of METTL3 substantially shortened the half-life of *Spry1* mRNA, suggesting that METTL3-mediated m^6^A modification within the CDS enhances the *Spry1* mRNA stability. The increased *Spry1* expression observed in *Adap*^*−/−*^ macrophages is due to the elevated m^6^A levels in *Spry1* mRNA transcripts. The recognition of methylated RNA by m^6^A readers is a key step in regulating mRNA stability, expression, and function. Typically, m^6^A-modified mRNAs are recognized and regulated by YTHDFs or IGF2BPs, which act as direct m^6^A readers [9, 35–37]. Our data demonstrate that IGF2BP2, but not IGF2BP1 or IGF2BP3, specifically binds to m^6^A-modified *Spry1* mRNA in a METTL3-dependent manner. Furthermore, knockdown of IGF2BP2, but not IGF2BP1/3, directly impaired LPS-induced *Spry1* mRNA stability. Thus, our findings demonstrate that METTL3 stabilizes *Spry1* mRNA through an m^6^A-IGF2BP2-dependent mechanism. The enhanced SPRY1 expression in *Adap*^*−/−*^ macrophages exacerbates the inflammatory response by activating the NF-κB pathway, which is in line with the previous reports demonstrating that SPRY1 promotes CXCL12 expression in tumor cells via activating the NF-κB signaling pathway, independently of the ERK pathway [27]. Additionally, S*pry1* knockout mice exhibit impaired NF-κB pathway activation and reduced nuclear translocation of p65 following LPS injection [38]. However, it remains to be seen exactly how the ADAP upregulates the m^6^A modification of *Spry1* mRNA. One plausible explanation is that ADAP binding to METTL3 may restrain its enzymatic activity or hinder the recruitment of enzymes involved in m^6^A modification to the vicinity of METTL3. A similar regulatory model has been reported in embryonic stem cells, where ZFP217 interacts with and sequesters METTL3 protein, fine-tuning m^6^A deposition by downregulating methylase activity [39]. Investigation of such assumptions in detail would be of interest for future study.

**Fig. 8:**
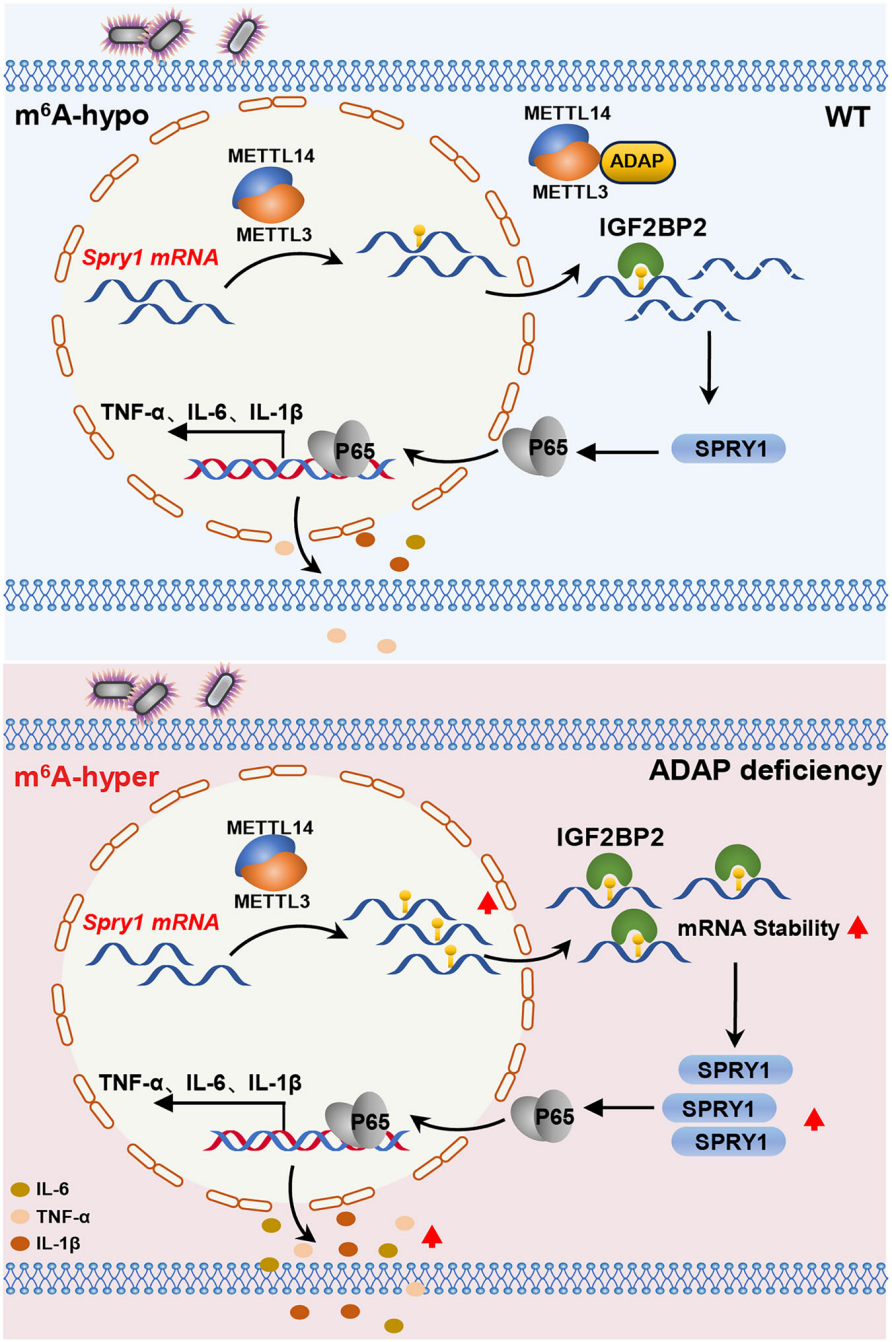
Schematic model of the ADAP-METTL3 module regulating macrophage inflammatory responses during sepsis through m^6^A modification of *Spry1.* Our data demonstrate that LPS stimulation enhances both m^6^A methylation and expression levels of *Spry1* mRNA in macrophages in a METTL3-dependent manner. m^6^A-modified *Spry1* mRNAs within the 3’-UTR are selectively recognized by IGF2BP2, which stabilizes *Spry1* transcripts. This resulted in an amplification of the production of pro-inflammatory cytokines such as TNF-α, IL-1β, and IL-6, through NF-κB pathway activation in macrophages. When ADAP is under-expressed, LPS-induced m^6^A methylation of *Spry1* mRNA is further augmented, resulting in a pronounced increase in pro-inflammatory cytokine secretion. Together, these results define a novel ADAP-METTL3 regulatory axis that fine-tunes macrophage inflammatory responses via m^6^A-dependent post-transcriptional control of *Spry1*.

In summary, we propose a model for the functional role of the ADAP-METTL3 module in controlling the inflammatory response in macrophages during sepsis via m^6^A modification of *Spry1* (Fig. 8). We show that LPS stimulation enhances both m^6^A methylation and expression levels of *Spry1* mRNA in macrophages, which is METTL3-dependent. The methylated *Spry1* mRNAs containing m^6^A modification in the CDS is recognized by insulin-like growth factor 2 mRNA-binding protein 2 (IGF2BP2), resulting in an enhanced stability of *Spry1* mRNA and an increased production of pro-inflammatory cytokines via activation of NF-κB pathway in macrophages. When ADAP is under-expressed, LPS-induced *Spry1* mRNA level in m^6^A methylation in macrophages is further potentiated, leading to an enhanced production of pro-inflammatory cytokines. Together, our findings highlight a novel role for ADAP-METTL3 in the control of the inflammatory response in macrophages via *Spry1* m^6^A modification, which indicates that effectively and specifically activating the TLR4-METTL3-ADAP-SPRY1 signaling axis may represent a promising target for immunotherapies in inflammatory diseases such as sepsis.

## Materials and methods

### Antibodies and reagents

Lipopolysaccharide (LPS, 055:B5) was purchased from Sigma-Aldrich, and STM2457 from Med Chem Express (MCE, HY-134836). The antibodies used included: anti-ADAP (Cell Signaling Technology (CST, #11957), anti-METTL3 (Proteintech, 15073-1-AP), anti-METTL14 (Sigma-Aldrich, HPA038002), anti-m^6^A (Proteintech, 68055-1-Ig), anti-SPRY1 (CST, 13013), anti-IGF2BP1 (Proteintech, 22803-1-AP), anti-IGF2BP2 (Proteintech, 11601-1-AP), anti-IGF2BP3 (Proteintech, 14642-1-AP), anti-FTO (Proteintech, 27226-1-AP), anti-ALKBH5 (Proteintech, 16837-1-AP), anti-WTAP (Proteintech, 60188-1-Ig), anti-β-actin (Proteintech, 66009-1-Ig), anti-p65 (Proteintech, 10745-1-AP), anti-Phospho-NF-κB-p65 (Ser536) (CST, 3033), anti-HA (Proteintech, 51064-2-AP), and anti-FLAG (Proteintech, 20543-1-AP). HRP-linked anti-rabbit and anti-mouse IgG antibodies were obtained from CST (7074 and 7076, respectively). Flow cytometry antibodies included APC anti-mouse CD206 (BioLegend, C068C2), PE-Cy7 anti-mouse CD86 (BD Biosciences, 560582), PE anti-mouse F4/80 (BD Biosciences, 565410), and FITC anti-mouse CD11b (BD Biosciences, 553310), with corresponding IgG isotypes from Biosciences.

### Cell culture

Peritoneal macrophages (PMs) were isolated from mice via peritoneal lavage using 5 ml of phosphate-buffered saline (PBS), 3 days after injection of 3% thioglycolate. Bone marrow-derived macrophages (BMDMs) were obtained by flushing the femurs and tibias of mice with PBS using a syringe, followed by filtration through a 70-μm mesh filter to remove debris. The BMDMs were cultured in RPMI 1640 medium supplemented with 10% fetal bovine serum (FBS), 20% L929-conditioned medium, 100 U/ml penicillin/streptomycin, 2 mM L-glutamine, and 50 µM β-mercaptoethanol for 7 days. Immortalized BMDMs were prepared as previously described [22]. HEK 293T and RAW 264.7 cells were purchased from the American Type Culture Collection (ATCC) and maintained in high-glucose DMEM supplemented with 10% FBS and 100 U/ml penicillin-streptomycin.

### Animals

*Adap*^*−/−*^ mice were originally generated by Prof. Peterson and colleagues as described and kindly provided by Dr. CE Rudd (University of Cambridge, UK) [40]. Wild-type (WT) C57BL/6, *Lyz2-Cre* transgenic mice (Cat#: T003822), and *Mettl3*^*flox/flox*^ mice (*Mettl3*^*f/f*^) (Cat#: T006659) bearing LoxP sites were purchased from Gempharmatech Co. Ltd. (Nanjing, China). To generate macrophage-specific METTL3 knockout mice, *Mettl3*^*f/f*^ mice were crossed with *Lyz2-Cre* transgenic mice.

*Mettl3*^*f/f*^*Lyz2-Cre*^*+/−*^*Adap*^*−/*^^−^ mice were obtained by crossing *Mettl3*^*f/f*^*Lyz2-Cre*^*+/−*^ mice with *Adap*^*−/−*^ mice. Genotypes were identified by allele-specific PCR with primers listed in Supplementary Table 1. All mice were of the C57BL/6 genetic background. Sex-matched littermate mice (6–8 weeks) were used in all experiments. Mice were bred and maintained under specific pathogen-free conditions at the animal facility at Soochow University. According to the randomization principle, mice of each genotype were randomly and equally divided into two groups before the experiment: the control group and the model group. For the LPS-induced endotoxemia model, eight-week-old mice were intraperitoneally injected with saline or 20 mg/kg LPS for 24 h. For the cecal ligation puncture (CLP) sepsis model, surgery was performed as described previously [41]. Briefly, the cecum was isolated and ligated below the ileocecal valve and 1 cm from the end of the cecum, and then punctured once with a 21-G needle, followed by extrusion of a small drop of fecal contents. The cecum was then returned to the peritoneal cavity, and the skin layer was stitched shut using medical-surgical clips. In sham-operated control mice, the cecum was exposed, but no ligation nor puncture was performed. All animal procedures were approved by the Ethics Committee of Soochow University (ECSU) (Approved No 202409A0495).

### RNA extraction and real-time quantitative polymerase chain reaction (RT-qPCR)

Total RNA was extracted from cells using TRIzol reagent (Invitrogen, Carlsbad, CA, USA). RNA quality and concentration were assessed using a Biotek Synergy. Reverse transcription of 1 μg RNA was performed using Hifair Ⅲ 1st Strand cDNA Synthesis Supermix (YEASEN, 11141ES60, China) according to the manufacturer’s instructions. RT-qPCR analysis was conducted on an Eppendorf Realplex using Hieff UNICON Universal Blue qPCR SYBR Green Master Mix (YEASEN, 11184ES25, China). Primers for qPCR analysis were listed in Supplementary Table 2, derived from Origene and synthesized by Sangon Biotech.

### Immunoblotting and immunoprecipitation

Cells were harvested, and proteins were extracted using a lysis buffer (1% Triton X-100 (v/v) in 20 mM Tris-HCl (pH 8.3), 150 mM NaCl) supplemented with a protease and phosphatase inhibitor cocktail, incubated on ice for 30 min. The samples were centrifuged at 12,000 *×* *g* for 15 min to remove debris, and the supernatant was mixed with 5 × sodium dodecyl sulfate polyacrylamide gel electrophoresis (SDS-PAGE) loading buffer and boiled for 5 min. Protein lysates were separated using SDS-PAGE, followed by transferring onto nitrocellulose membranes (Millipore). The membranes were blocked with 5% nonfat milk powder in 0.1% phosphate-buffered solution with 0.05% Tween-20 (PBST) for 2 h at room temperature and then incubated with primary antibodies at 4 °C overnight. After washing with PBST, the membranes were incubated with HRP-conjugated secondary antibodies for 1 h at room temperature, developed with enhanced chemiluminescence, and the membranes were visualized using a Tanon 4600.

For the immunoprecipitation assay, the cell lysates were incubated with primary antibodies for 3 h at 4 °C. The protein-antibody mixture was then mixed with Protein A/G PLUS-Agarose (GE Healthcare) and further incubated for 12 h at 4 °C. After washing with the lysis buffer three times, the agarose beads were boiled with 2 × SDS-PAGE loading buffer and the associated proteins were detected by immunoblotting.

### Flow cytometry analysis

BMDMs were harvested and resuspended in FCSA buffer (PBS with 2% FBS) after treatment with PBS or LPS. Fluorescence-conjugated antibodies anti-CD11b, anti-F4/80, anti-CD86, and the corresponding IgG isotypes were added to the cells in FASC buffer and incubated on ice for 1 h. For intracellular CD206 staining, fixed and permeabilized cells were treated using the eBioscience Intracellular Fixation & Permeabilization Buffer Set (Thermo Fisher Scientific, 88-8824-00) according to the manufacturer’s instructions. The cells were then washed twice and resuspended in PBS. Stained cells (1 × 10^4^) were analyzed using flow cytometry on a FACS Calibur.

### Hematoxylin-eosin (H&E) staining

Lungs, livers, and left kidneys from mice were fixed in 4% paraformaldehyde, dehydrated, and embedded in paraffin. Hematoxylin and eosin staining was performed on 5-μm sections as described previously [42]. Stained slides were examined under a microscope (Nikon, Tokyo, Japan). The H&E score of lung lesions was assessed based on the extent of epithelial denaturation/collapse, pneumocyte degeneration, inflammatory cell infiltration, edema, hemorrhage, exudation, and parenchymal wall expansion, scored 0 (normal) to 4 (severe) per parameter [43]. Liver injury was evaluated using Suzuki’s criteria [44], scoring vascular congestion, hepatocyte vacuolization, and necrosis from 0 (none) to 4 (severe), with the sum generating a composite score (0–12). Kidney injury was scored 0–5 according to tubular damage percentage: 0 (normal), 1 (focal necrosis), 2 (≤25%), 3 (25–50%), 4 (51–75%), and 5 (≥76%) [45]. The tissue injury scores were evaluated by two pathologists who were blinded to the sample identities.

### Enzyme-linked immunosorbent assay (ELISA)

The concentrations of TNF-α, IL-6, and IL-1β in mouse serum were detected by ELISA kits, according to the manufacturer’s instructions (R&D Systems).

### M^6^A quantification

The m^6^A levels of RNA in cells were quantified by dot blot assays. Total RNA was extracted from cells using Trizol (Invitrogen, USA) reagent and measured using a Biotek Synergy. For the dot blot assay, the obtained RNA was diluted to 250 ng/μl and denatured at 95 °C for 5 min, after which 2 μl of the diluted RNA was separately spotted on the Hybond-N^+^ membrane (Millipore, USA). The membranes were then UV cross-linked at 245 nm for 1 h and blocked with 5% nonfat milk at room temperature for 1 h. The membranes were then incubated with anti-m^6^A antibody (68055-1-Ig, Proteintech, 1:1000) overnight at 4 °C. After washing three times in PBST, horseradish peroxidase (HRP)-conjugated secondary antibody (1:10,000 dilution) was added to the membranes for 1 h at room temperature. After washing with TBST three times, signals were detected using the Tanon system. Membranes were stained in a 0.02% methylene blue solution (pH 5.2) to show the total RNA for each group, and the results were captured by a camera.

### Methylated RNA immunoprecipitation sequencing (MeRIP-seq) and RNA-seq

PMs isolated from WT and *Adap*^−/−^ mice were cultured in 10 cm dishes and treated with 1 μg/ml LPS for 6 h. Total RNA was extracted and purified with TRIzol reagent (Invitrogen, USA) following the manufacturer’s procedure. The RNA amount and purity were examined using a Nanodrop 2000 (Thermo Fisher Scientific, USA). Subsequently, the RNA was fragmented into approximately 100-nt fragments using RNA Fragmentation Reagents (Invitrogen, AM8740). Approximately one-tenth of the fragmented RNA was preserved as the input control for further RNA sequencing. The remaining RNA was incubated with an m^6^A-specific antibody (Synaptic Systems, 202203), and immunoprecipitation was performed using an EpiMark N6-Methyladenosine Enrichment Kit (New England Biolabs) following the manufacturer’s protocols. Both MeRIP-seq and RNA-seq were performed on the Illumina HiSeqTM 2500/4000 by Gene Denovo Biotechnology Co., Ltd (Guangzhou, China), and bioinformatic analysis was conducted using Omicsmart, a real-time interactive online platform for data analysis (http://www.omicsmart.com).

### MeRIP-qPCR

The protocol of MeRIP was adapted from previous descriptions with minor modifications [46, 47]. Briefly, 20 μg of total RNA was isolated and fragmented into segments of 100 nucleotides or less using a fragmentation buffer (Invitrogen, AM8740); one-tenth of the fragmented RNA was retained as an input control. Then, protein A/G magnetic beads (Thermo Fisher Scientific, 88802) were prewashed and incubated with 5 μg of anti-m^6^A antibody (Proteintech, 68055-1-Ig) or anti-mouse immunoglobulin G (IgG) at 4 °C overnight with rotation. After three washes, the antibody-conjugated beads were combined with the remaining fragmented RNA in immunoprecipitation buffer (150 mM NaCl, 10 mM Tris-HCl, pH 7.4, 0.1% NP-40) containing RNase inhibitors and incubated for 6 h at 4 °C. After three washes, TRIzol was added to the beads-antibody-RNA complexes to extract the bound RNA. Genes with m^6^A modifications were identified using RT-qPCR with specific primers, and the corresponding m^6^A enrichment was calculated by normalizing to the input control.

### RIP-qPCR

Cells were lysed in 1.28 M sucrose, 40 mM Tris-HCl (pH 7.5), 20 mM MgCl_2_, and 4% Triton X-100 with protease inhibitor added, for 20 min on ice, followed by centrifugation at 2500 × *g* for 15 min. The precipitate was collected in RIP buffer (150 mM KCl, 25 mM Tris pH 7.4, 5 mM DTT, 0.5% NP-40) supplemented with 100 U/ml RNase and protease inhibitors. Following centrifugation at 13,000 × *g* for 10 min, the supernatant was incubated with IGF2BP1, IGF2BP2, IGF2BP3, or the corresponding IgG antibodies for 5 h, and protein A/G magnetic beads were then added and incubated for an additional 3 h at 4 °C. After washing three times with RIP buffer, RNA was eluted using TRIzol methods. An RT-qPCR assay was performed to detect RNA binding to proteins or antibodies.

### siRNA transfection

METTL3 siRNA (si-METTL3), IGF2BP1 siRNA (si-IGF2BP1), IGF2BP2 siRNA(si-IGF2BP2), IGF2BP3 siRNA (si-IGF2BP3), SPRY1 siRNA (si-SPRY1), and related negative control siRNA (si-NC) were all synthesized by Sangon Biotech (Shanghai, China). WT and ADAP knockdown RAW 264.7 cells were transfected with siRNA using Lipofectamine RNAiMAX reagent (13778030, Thermo Fisher Scientific, USA) when the cell confluence reached ~80%. Cells were collected 24–48 h post-transfection for further experiments. All siRNA sequences are listed in Supplementary Table 3.

### Plasmid constructs and mutagenesis

HA-tagged ADAP and its mutant were previously described [21]. FLAG-METTL3 (P8770), FLAG-METTL14 (P22487), and HIS-IGF2BP2 (P41751) were purchased from MiaoLingBio (China), MYC-WTAP (HG12018) was obtained from SinoBiological. Truncated FLAG-METTL3 mutants were generated by deleting the sequence encoding amino acids 389–581 or 1–389 using the FLAG-METTL3 plasmid as a template. D394A (GAC to GCC) and W397A (TGG to GCG) mutations in FLAG-METTL3 were introduced with a Hieff Mut™ Site-Directed Mutagenesis Kit (YEASEN, 11003ES10, China) following the manufacturer’s instructions. For luciferase assays, a fragment containing the m^6^A site in *Spry1* cDNA was cloned downstream of the pTA-LUC firefly luciferase promoter (Clontech) to create *Spry1* wild-type (*Spry1*-WT). A mutated version, *Spry1*-Mut, was generated by altering the m^6^A motif (5’-GGACT-3’) to 5’-GGTCT-3’ using the Mutagenesis Kit above. Each plasmid insert was confirmed by Sanger sequencing.

### Lentiviral plasmids and cell transduction

Lentiviral plasmids pLVX-METTL3, pLV2-IGF2BP2-shRNA1-EGFP, pLV3-CMV-IGF2BP2-3 × FLAG-CopGFP, and pLV3-CMV-SPRY1-3 × FLAG-CoGFP were constructed by MiaoLingBio. Packaging plasmids were co-transfected with these lentiviral vectors into HEK 293T cells. Viral supernatants were harvested 48 h post-transfection and used to infect WT or ADAP knockdown RAW 264.7 cells with polybrene (8–10 µg/ml). Knockdown efficiency was validated by Western blot analysis.

### Luciferase reporter assay

Dual-Luciferase assays were performed by a Dual-Luciferase Reporter Gene Assay Kit (YEASEN, 11402ES60) according to the manufacturer’s protocol. HEK 293T cells were seeded in six-well plates and co-transfected with IGF2BP2, METTL3-WT or METTL3-Mut, *Spry1*-WT, or *Spry1*-Mut plasmids, along with 0.5 μg pRL-TK Renilla luciferase reporter, using DNA Transfection Reagent (40802ES03, YEASEN). After 48 h, cells were lysed, and firefly luciferase (FLuc) activity was normalized to Renilla luciferase (RLuc).

### Immunofluorescence staining

PMs isolated from WT and *Adap*^*−/−*^ mice were seeded on glass slides in 12-well plates. After PBS or LPS treatment for 6 h, cells were fixed with 4% paraformaldehyde (PFA) for 15 min and permeabilized with 0.25% Triton X-100 for 15 min. Cells were washed three times with PBS and blocked with 5% goat serum for 1 h, followed by overnight incubation with primary antibodies at 4 °C. After washing with PBS three times, secondary antibodies were added and incubated at room temperature for 1 h. Nuclei were stained with 4′,6′­diamidino­-2­-phenylindole dihydrochloride (DAPI, Molecular Probes, Beyotime Biotechnology, Shanghai, China) for 5 min. Slides were imaged with a Zeiss LSM 880 confocal microscope. The primary antibodies used in this work included ADAP (1:500, BD Biosciences, 610944, USA) and METTL3 (1:1000, Proteintech, 15073-1-AP, USA). Secondary antibodies included goat anti-mouse IgG Alexa Fluor 488 (1:1000, Invitrogen, A-11001, USA), goat anti-rabbit IgG Alexa Fluor 568 (1:1000, A-11011, Invitrogen, USA).

### RNA stability assay

RNA stability was analyzed by treating cells with 5 μg/ml actinomycin D (ActD), followed by cell collection at specific time intervals. Total RNA was extracted and subjected to RT-qPCR to quantify relative *Spry1* mRNA abundance (relative to 0 h).

### Statistical analysis

All data are expressed as the mean ± standard error of the mean (SEM) from at least three independent experiments. For statistical analysis, unpaired two-tailed Student’s *t*-test and comparisons among more than two groups were performed using ANOVA. For data satisfying homogeneity of variance, within-group multiple comparisons were conducted with the Bonferroni correction. If heterogeneity of variance was detected, Welch’s adjusted ANOVA was employed. Statistical analysis was performed with GraphPad Prism 8.0. *P ≤ 0.05, **P ≤ 0.01, and ***P ≤ 0.001 were considered statistically significant.

## Supplementary information

supplementary figure1

supplementary figure2

supplementary figure3

supplementary figure4

supplementary figure5

supplementary figure6

Supplementary Figure Legends

Supplementary Tables

Original western blots

## Data Availability

The data supporting the findings of this study are available from the corresponding author upon reasonable request. The MeRIP-seq data generated in this work have been deposited in the Gene Expression Omnibus (GEO) database (GEO accession number: GSE302809). All other relevant data are available from the corresponding author upon request.
