# Supplementary figures and images for: ADAP-METTL3 modulates the inflammatory responses of macrophages via m6A modification of Spry1
Source: Cell Death Dis. 2025 Oct 7;16(1):708. doi: 10.1038/s41419-025-08008-x (PMC12504520; doi:10.1038/s41419-025-08008-x)

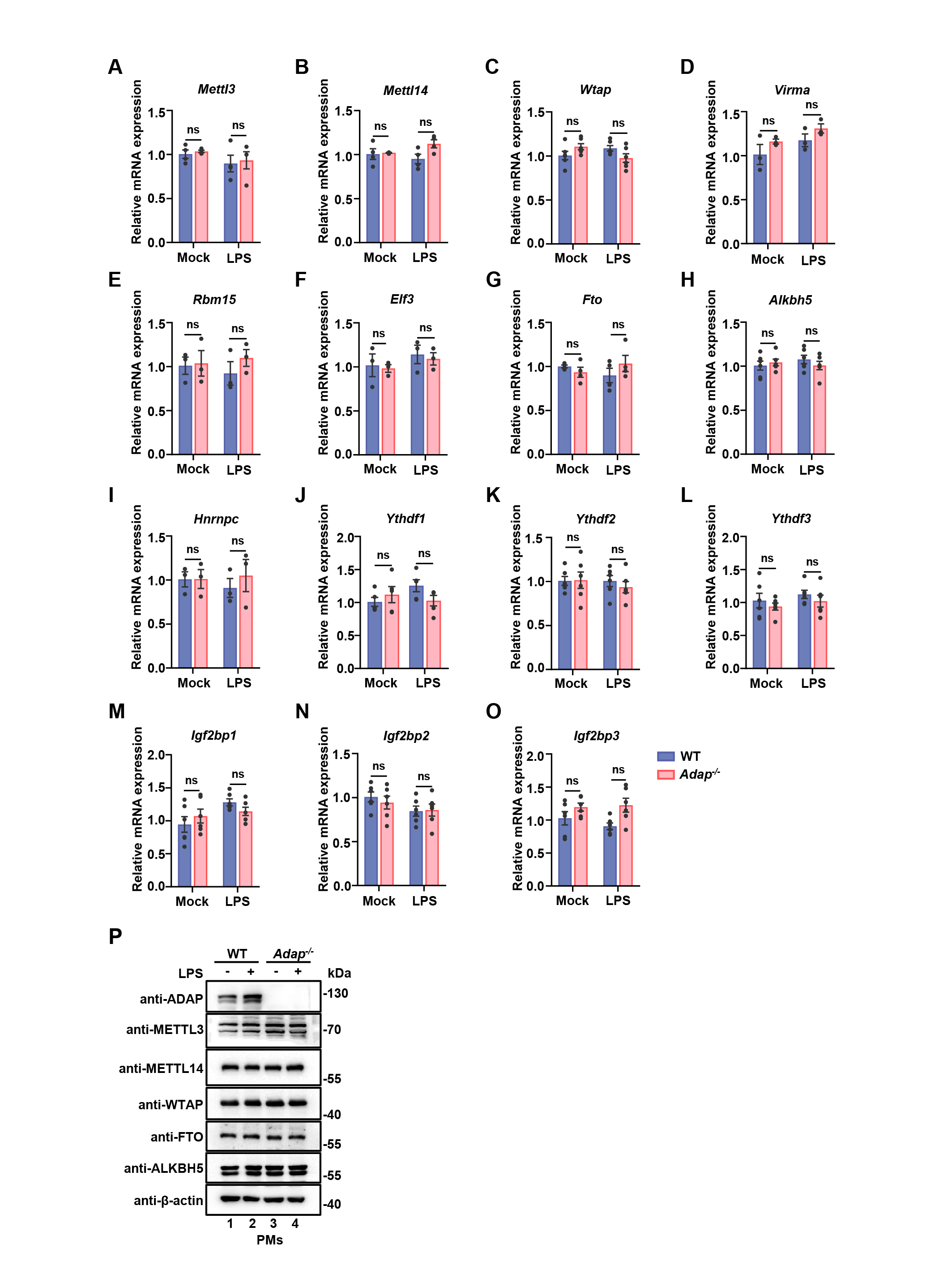

Supplement: Supplementary file 1 — supplementary figure1 [file 41419_2025_8008_MOESM1_ESM.png]

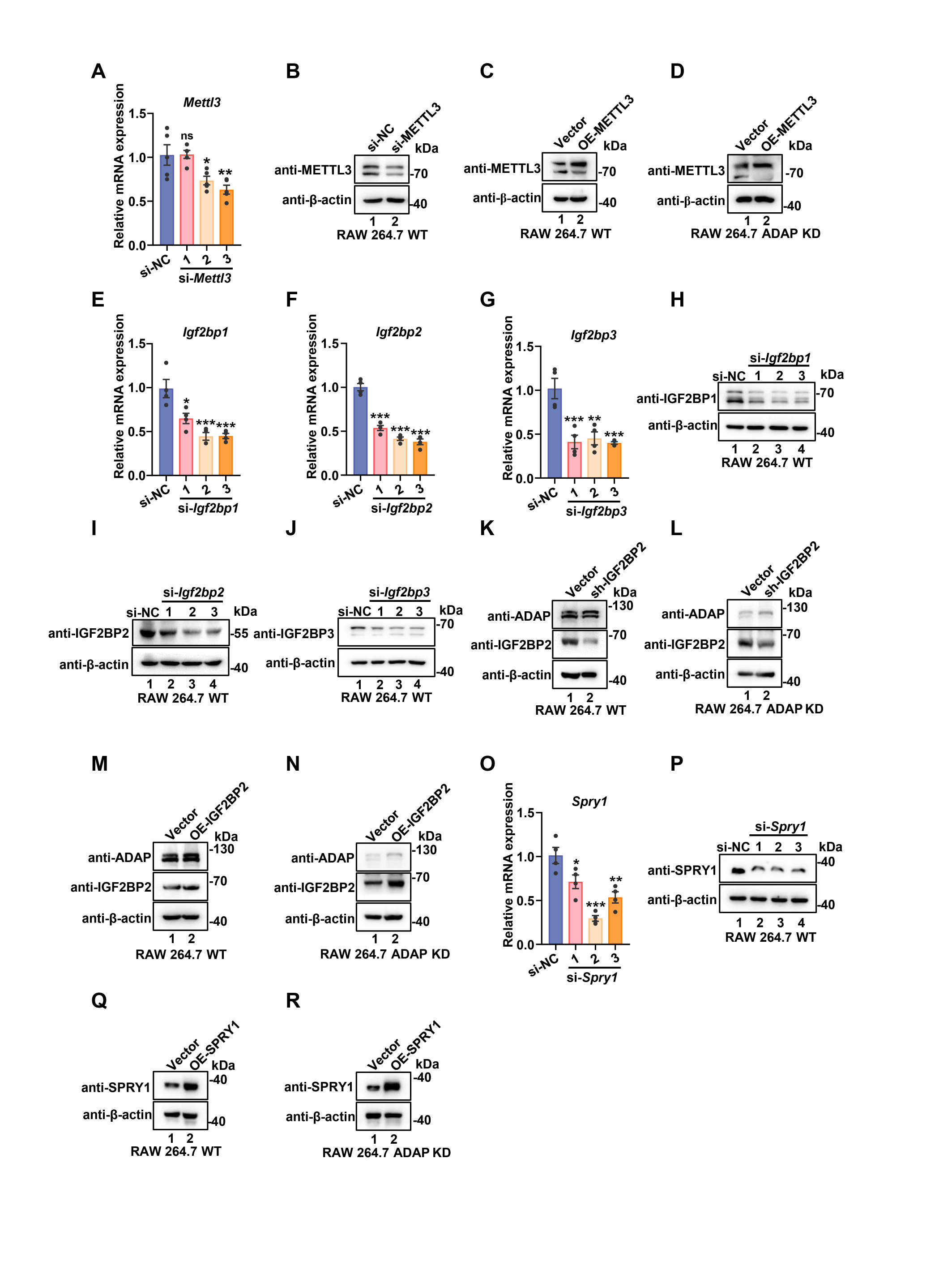

Supplement: Supplementary file 2 — supplementary figure2 [file 41419_2025_8008_MOESM2_ESM.png]

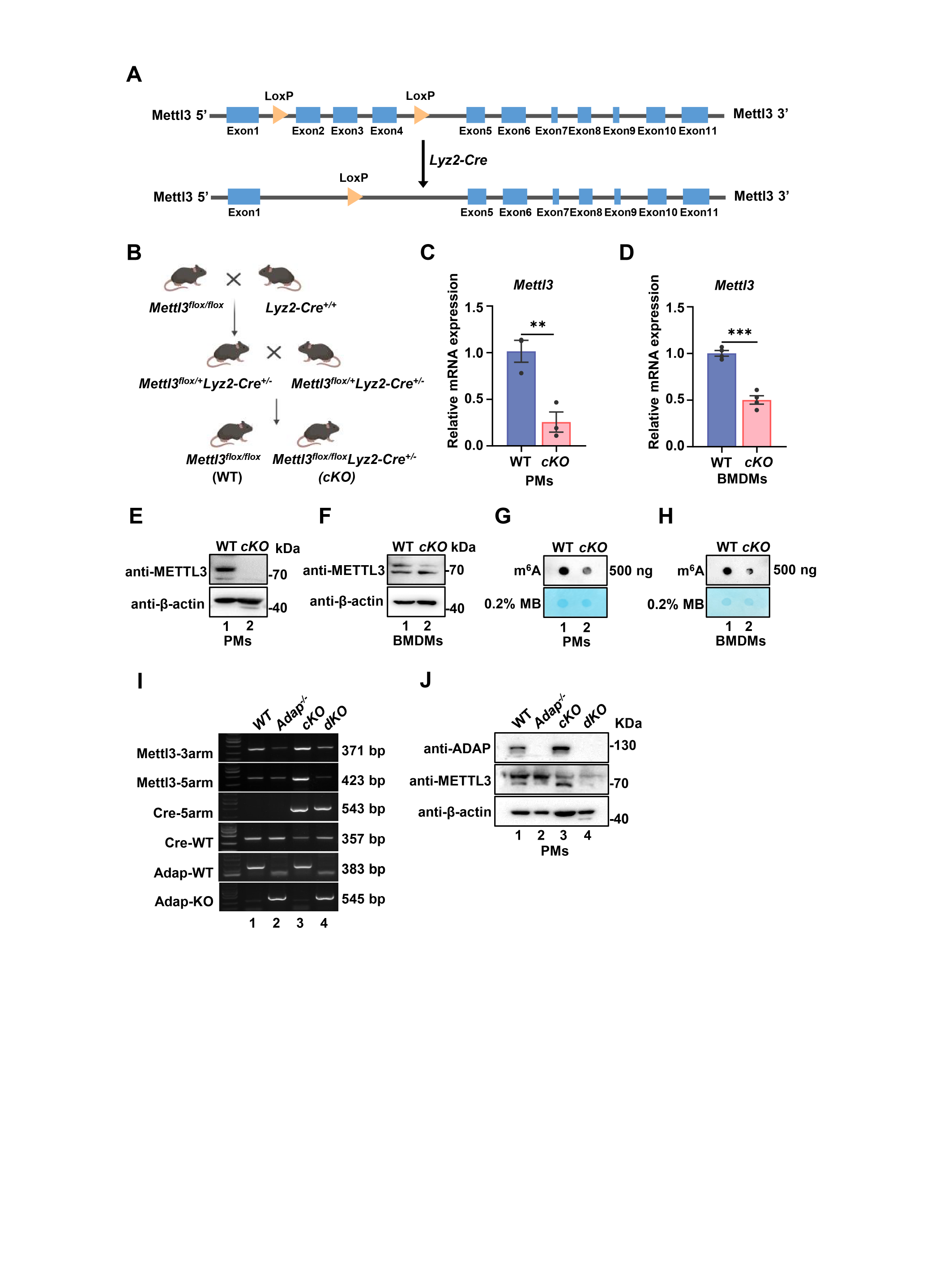

Supplement: Supplementary file 3 — supplementary figure3 [file 41419_2025_8008_MOESM3_ESM.png]

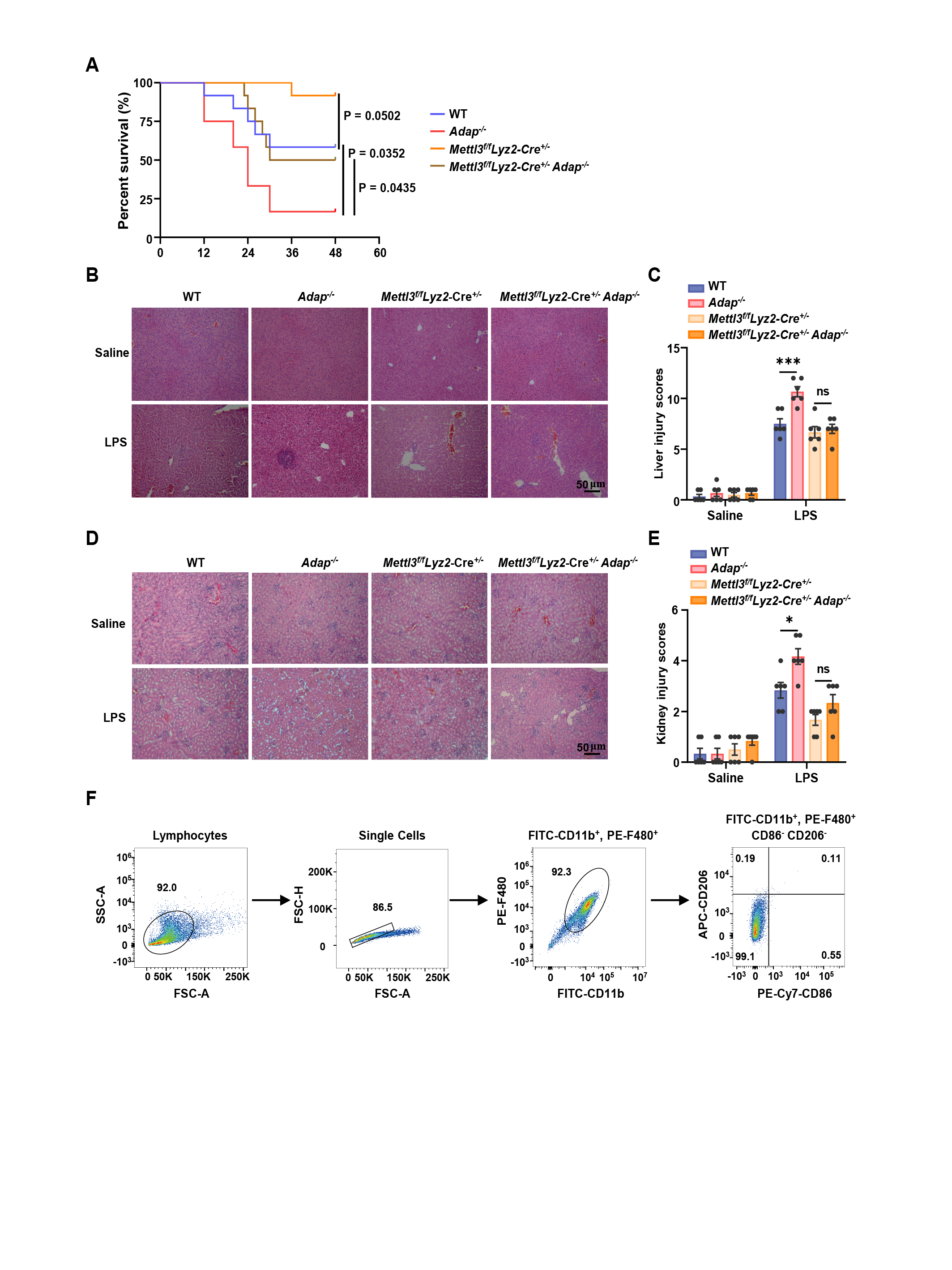

Supplement: Supplementary file 4 — supplementary figure4 [file 41419_2025_8008_MOESM4_ESM.png]

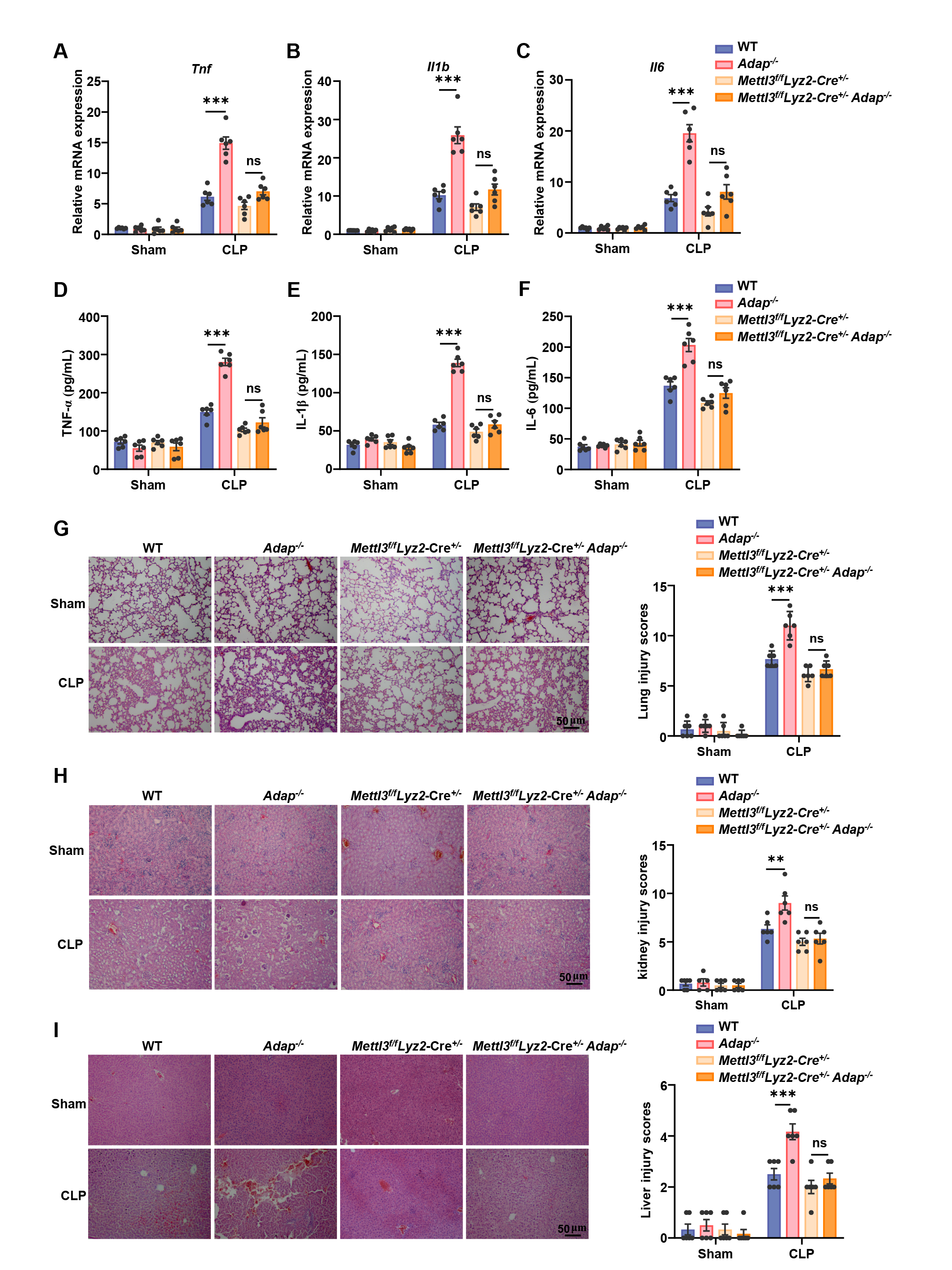

Supplement: Supplementary file 5 — supplementary figure5 [file 41419_2025_8008_MOESM5_ESM.png]

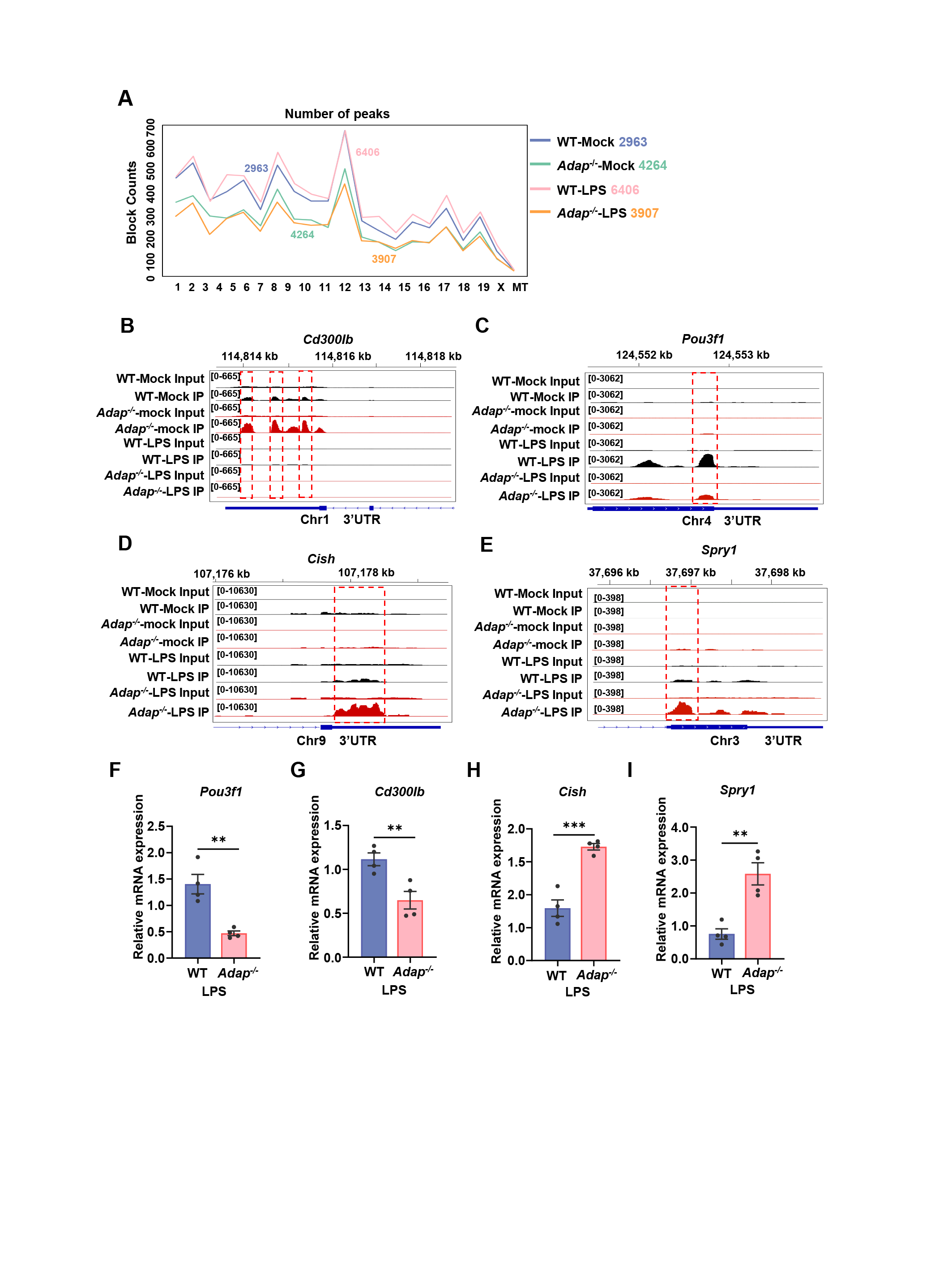

Supplement: Supplementary file 6 — supplementary figure6 [file 41419_2025_8008_MOESM6_ESM.png]
