# Supplementary material for: ADAP-METTL3 modulates the inflammatory responses of macrophages via m6A modification of Spry1
Source: Cell Death Dis. 2025 Oct 7;16(1):708. doi: 10.1038/s41419-025-08008-x (PMC12504520; doi:10.1038/s41419-025-08008-x)
Supplement: Supplementary file 7 — Supplementary Figure Legends [file 41419_2025_8008_MOESM7_ESM.docx]

**Supplementary Information**

**Figure S1. The absence of ADAP does not affect LPS-induced METTL3 expression in macrophages**

**A-O,** PMs isolated from WT and *Adap^-/-^* mice were either mock-treated or stimulated with LPS(1 μg/ml) for 6 h. Total RNA were extracted, and the mRNA expression of key m^6^A regulatory genes, including *Mettl3* (A), *Mettl14* (B), *Wtap (C)*, *Virma (D)*, *Rbm15* (E), *Elf3* (F), *Fto* (G), *Alkbh5* (H), *Hnrnpc* (I), *Ythdf1* (J), *Ythdf2* (K), *Ythdf3* (L), *Igf2bp1* (M), *Igf2bp2* (N), and *Igf2bp3* (O), were quantified using RT-qPCR. The relative mRNA levels were normalized to the *Gapdh* expression. n = 4. ns, nonsignificant. **P,** PMs from WT or *Adap^-/-^* mice were either mock-treated or treated with LPS (1 μg/ml, 12 h) as indicated. Cells were collected and lysed, and protein levels of METTL3, METTL14, WTAP, FTO, and ALKBH5 were analyzed by immunoblotting. β-actin was served as a loading control.

**Figure S2.** **Verification of knockdown and overexpression in cell lines.**

**A,** RAW 264.7 cells were transfected with either a negative control siRNA or three different *Mettl3*-targeting siRNAs. 24 h after transfection, total RNA was extracted, and the mRNA levels of METTL3 were quantified using RT-qPCR. The relative expression of METTL3 was normalized to the housekeeping gene *Gapdh*. n = 5. **P* < 0.05, ***P* < 0.01, ns, nonsignificant, compared versus non-targeting control siRNA by Student’s *t* test. **B,** RAW 264.7 cells were transfected with either a control scrambled siRNA or METTL3 siRNA for 48 h. Cell lysates were prepared and subjected to immunoblotting using an anti-METTL3 antibody. **C-D,** WT(C) and ADAP knockdown (D) RAW 264.7 cells were infected with either lentivirus vector or a lentivirus containing METTL3-overexpression sequence (OE) for 48 h. Following puromycin selection for 14 days, cells were harvested, and whole-cell lysates were analyzed by Western blotting using antibodies against the indicated proteins. **E-G,** RAW 264.7 cells were transfected with *Igf2bp1, Igf2bp2, or Igf2bp3* siRNA, or a non-targeting control siRNA for 24 h. The mRNA levels of *Igf2bp1* (E), *Igf2bp2* (F) and *Igf2bp3* (G) were quantified by RT-qPCR. n = 4 for each group. *P* values were calculated by Student’s *t* test. **P* < 0.05, ***P* < 0.01, ****P* < 0.001 compared versus non-targeting control siRNA. **H-J,** RAW 264.7 cells were transiently transfected with *Igf2bp1, Igf2bp2, or Igf2bp3* siRNA, or a non-targeting control siRNA. 48 h post transfection, cells were lysed, and the lysates were analyzed by immunoblotting with anti-IGF2BP1(H), anti-IGF2BP2(I), and anti-IGF2BP3 antibody (J). β-actin was used as an internal control. **K and L,** WT RAW 264.7 cells (K) and ADAP knockdown RAW 264.7 cells (L) were infected with lentivirus carrying either a control vector plasmid or a shRNA targeting IGF2BP2. Following puromycin selection for 7–14 days, cells were harvested and lysed, and the knockdown efficiency of IGF2BP2 was assessed by Western blotting. **M and N,** WT RAW 264.7 cells (M) and ADAP knockdown RAW 264.7 cells (N) were infected with lentivirus containing either a control vector plasmid or a specific plasmid overexpressing IGF2BP2. 48 h post-infection, cells were cultured in the presence of puromycin for 7–14 days. Cell lysates were then analyzed by immunoblotting using the indicated antibodies. **O,** RAW 264.7 cells were transfected with either a non-targeting control siRNA or *Spry1-*targeting siRNA for 24 h. Total RNA was isolation, and the mRNA expression of *Spry1* was quantified by RT-qPCR. n = 4. The student’s *t* test was used to determine statistical significance. **P* < 0.05, ***P* < 0.01, ****P* < 0.001 compared versus non-targeting control siRNA. **P,** RAW 264.7 cells were transfected with *Spry1*-targeting siRNA or a non-targeting control siRNA. Forty-eight hours post-transfection, cells were lysed, and the lysates were subjected to immunoblotting with the indicated antibodies. **Q and R,** WT RAW 264.7 cells (Q) and ADAP knockdown RAW 264.7 cells (R) were infected with either a lentivirus vector or a lentivirus carrying a specific plasmid overexpressing *Spry1* for 48 h, followed by puromycin selection for 7–14 days. The expression of SPRY1 was assessed by Western blotting in cell lysates.

**Figure S3. Construction and verification of myeloid *Mettl3*-deficient mice.**

**A,** Schematic representation of the targeting strategy for generating *Mettl3* conditional knockout (*cKO*) mice**.** The floxed-*Mettl3* allele was engineered by flanking exons 2 and 4 with the loxP sites. Macrophage-specific deletion of *Mettl3* was achieved by crossing floxed-*Mettl3* mice with transgenic mice expressing Cre recombinase under the control of the lysozyme 2 (*Lyz2*) enhancer/promoter. **B,** Breeding strategy for generating *Mettl3* *cKO* mice. Floxed-*Mettl3* mice were crossed with *Lyz2-Cre* transgenic mice to produce *Mettl3^fl/fl^Lyz2-Cre^+/-^* (*cKO*) offspring. **C and D,** PMs (C) and BMDMs (D) were isolated from *Mettl3^fl/fl^* (WT) and *Mettl3^fl/fl^Lyz2-Cre^+/-^* (*cKO*) mice. Total RNA was extracted, and the mRNA expression levels of METTL3 were quantified by RT-qPCR. Relative expression values were normalized to *Gapdh* mRNA levels. Data are expressed as the mean ± SEM (n = 3). ***P* < 0.01, ****P* < 0.001 by unpaired Student’s *t* test. **E and F,** PMs (E) and BMDMs (F) from *Mettl3^fl/fl^* (WT) and *Mettl3^fl/fl^ Lyz2-Cre^+/-^* (*cKO*) mice were cultured and lysed. Cell lysates were subjected to immunoblotting with an anti-METTL3 antibody. β-actin was probed as a loading control. **G and H,** Total RNA was isolated from PMs (G) and BMDMs (H) of *Mettl3^fl/fl^* (WT) and *Mettl3^fl/fl^ Lyz2-Cre^+/-^*(*cKO*) mice. 500 ng mRNA was subjected to Dot blot analysis with an anti-m^6^A antibody. The levels of m^6^A are shown in the upper panel. Equal RNA loading was confirmed by methylene blue staining (lower panel). **I,** Genomic DNA was extracted from the tails of WT, *Adap^-/-^*, *Mettl3^f/f^Lyz2-Cre^+/-^* (*cKO*), and *Mettl3^f/f^Lyz2-Cre^+/-^Adap^-/-^* (*dKO*) mice. Genotyping was performed by genomic PCR. **J,** PMs from WT, *Adap^-/-^*, *Mettl3^f/f^Lyz2-Cre^+/-^* (*cKO*), and *Mettl3^f/f^Lyz2-Cre^+/-^Adap^-/-^* (*dKO*)mice were cultured and lysed. Cell lysates were analyzed by Western blotting using anti-ADAP and anti-METTL3 antibodies. β-actin was used as a loading control.

**Figure S4.** **METTL3 depletion ameliorates ADAP-deficiency-induced mortality and multi-organ damage in LPS-challenged mice.**

**A,** WT, *Adap*^-/-^, *Mettl3^f/f^Lyz2-Cre^+/-^*, and *Mettl3^f/f^Lyz2-Cre^+/-^Adap^-/-^* mice were

intraperitoneally injected with LPS (20 mg/kg), and survival was monitored over 24 h. Kaplan-Meier analysis was conducted (n = 12/group). **B and C,** WT, *Adap*^-/-^, *Mettl3^f/f^Lyz2-Cre^+/-^*, and *Mettl3^f/f^Lyz2-Cre^+/-^Adap^-/-^* mice were intraperitoneal injection saline or LPS (20 mg/kg) for 24 h. (B) Liver tissues were collected and subjected to hematoxylin and eosin (H&E) staining. Scale bars, 50 μm. (C) Liver injury was histologically scored and compared across groups (n = 6/group, two-way ANOVA). **D,** H&E staining of kidney sections from WT, *Adap*^-/-^, *Mettl3^f/f^Lyz2-Cre^+/-^*, and *Mettl3^f/f^Lyz2-Cre^+/-^Adap^-/-^* mice 24 h post-saline or LPS injection. Scale bar, 50 μm. **E,** Kidney scores were obtained from the pathological assessment of kidney tissues (n = 6). **F,** Representative flow cytometric gating strategy for FITC-CD11b^+^F4-80^+^CD86^+^ and FITC-CD11b^+^F4-80^+^CD206^+^ macrophage sub-populations. Cells were harvested from bone marrow-derived macrophages (BMDMs) of WT mouse. FSC, forward scatter; SSC, side scatter.

**Figure S5. METTL3 depletion ameliorates ADAP-deficiency-induced inflammatory damage in in septic CLP-treated mice.**

**A-C,** WT, *Adap*^-/-^, *Mettl3^f/f^Lyz2-Cre^+/-^*, and *Mettl3^f/f^Lyz2-Cre^+/-^Adap^-/-^* mice were subjected to CLP or sham operation for 24 h. Lung tissues were collected and total RNA was extracted. Relative mRNA expression levels of proinflammatory cytokines TNF-α (A), IL-1β (B) and IL-6 (C) were determined by RT-qPCR. (n = 6/group). *Gapdh* mRNA was used as an internal control to normalize the gene expression. ****P* < 0.001. ns, nonsignificant, two-way ANOVA. **D-F,** WT, *Adap*^-/-^, *Mettl3^f/f^Lyz2-Cre^+/-^*, and *Mettl3^f/f^Lyz2-Cre^+/-^Adap^-/-^* mice were subjected to CLP or sham operation for 24 h. Serum samples were subsequently collected and analyzed for concentrations of TNF-α (D), IL-1β (E), and IL-6 (F) using enzyme-linked immunosorbent assay (ELISA) (n = 5/group). Data are expressed as mean ± SEM. Statistical significance was determined using two-way ANOVA. ****P* < 0.001. ns, nonsignificant. **G-I,** WT, *Adap*^-/-^, *Mettl3^f/f^Lyz2-Cre^+/-^*, and *Mettl3^f/f^Lyz2-Cre^+/-^Adap^-/-^* mice were subjected to CLP or sham operation. At 24 h post operation, lung (G), kidney (H) and liver (I) tissues were collected, fixed in 4% paraformaldehyde, and processed for HE staining. Scale bars, 100 μm. Tissue injury scores were quantified as described in Methods.

**Figure S6. Validation of m^6^A-modified candidate genes.**

**A,** MeRIP-seq analysis was performed on PMs extracted from WT and *Adap*^-/-^ mice, either mock-treated or stimulated with LPS (1 μg/ml) for 6 h. The number of m^6^A peaks on each chromosome is presented in the graph. **B-E,** PMs from WT and *Adap^-/-^* mice were treated with or without 1 μg/ml LPS for 6 h. Total RNA was extracted and subjected to MeRIP-seq analysis. The m^6^A-methylated peaks of *Cd300lb* (B), *Pou3f1* (C), *Cish* (D) and *Spry1*(E) mRNA transcripts were visualized using Integrative Genomics Viewer (IGV) plots. **F-I,** PMs from WT and *Adap^-/-^* mice were stimulated with LPS (1 μg/ml) for 6 h. The mRNA expression levels of *Pou3f1* (F), *Cd300lb* (G), *Cish* (H), and *Spry1* (I) were quantified by RT-qPCR. n = 4. **P* < 0.05, ***P* < 0.01, ****P* < 0.001 compared versus WT group.
