# Supplementary material for: ADAP-METTL3 modulates the inflammatory responses of macrophages via m6A modification of Spry1
Source: Cell Death Dis. 2025 Oct 7;16(1):708. doi: 10.1038/s41419-025-08008-x (PMC12504520; doi:10.1038/s41419-025-08008-x)
Supplement: Supplementary file 8 — Supplementary Tables [file 41419_2025_8008_MOESM8_ESM.docx]

**Table S1. The primer sequence for mouse gene identification.**

| Names | Sequence (5’-3’) | |
| --- | --- | --- |
| Lyz2-Cre-5’ | Forward | AGTGCTGAAGTCCATAGATCGG |
|  | Reverse | CTGATTCTCCTCATCACCAGG |
| Lyz2-Cre-WT | Forward | AGTGCTGAAGTCCATAGATCGG |
|  | Reverse | GTCACTCACTGCTCCCCTGT |
| METTL3-5’ | Forward | ATAACCCTGGCTGTCCCG |
|  | Reverse | TCATTCACATGGCAGCACTT |
| METTL3-3’ | Forward | CCTTTGGAATGGCTACTGC |
|  | Reverse | ATCAGAAAGCCCATCCTCA |
| ADAP-WT | Forward | CCGTGGGGCCAAAGTCAGGAGAA |
|  | Reverse | CCCACCCCAAGGTCCTTTCTTAC |
| ADAP-KO | Forward | CCCACCCCAAGGTCCTTTCTTAC |
|  | Reverse | GCGCTACCGGTGGATGTGTGGAATGT |

**Table S2. The primer sequences for RT-qPCR.**

| Gene names | Sequence (5’-3’) | |
| --- | --- | --- |
| IL-1β | Forward | TGGACCTTCCAGGATGAGGACA |
|  | Reverse | GTTCATCTCGGAGCCTGTAGTG |
| IL-6 | Forward | TACCACTTCACAAGTCGGAGGC |
|  | Reverse | CTGCAAGTGCATCATCGTTGTTC |
| TNF-α | Forward | GGTGCCTATGTCTCAGCCTCTT |
|  | Reverse | GCCATAGAACTGATGAGAGGGAG |
| SPRY1 | Forward | GGACACTCAGCCTGCTACGATT |
|  | Reverse | AGCAGGTCTTCTCGCTACCGAA |
| IGF2BP1 | Forward | CCTGGCTCATAACAACTTCGTCG |
|  | Reverse | CCTTCACAGTGATGGTCCTCTC |
| IGF2BP2 | Forward | TGAAGCCTGTGCCAATGCTGAG |
|  | Reverse | CCAGTCGAAAAGATGCCAAGTGC |
| IGF2BP3 | Forward | CCACCCAGTTTGTTGGAGCCAT |
|  | Reverse | GGATAGTAATGGACTTCTCCGCG |
| METTL3 | Forward | CAGTGCTACAGGATGACGGCTT |
|  | Reverse | CCGTCCTAATGATGCGCTGCAG |
| POU3F1 | Forward | ACAGCCTGCAACTGGAGAAGGA |
|  | Reverse | CAGGCGCATAAACGTCGTCCAT |
| CISH | Forward | GGATCTGCTGTGCATAGCCAAG |
|  | Reverse | CTCGAACTAGGAATGTACCCTCC |
| CD300LB | Forward | ACGGCAGTACAGACATGGTGTC |
|  | Reverse | GAGTTGACCTCTTCAGCCAGAG |
| GAPDH | Forward | CATCACTGCCACCCAGAAGACTG |
|  | Reverse | ATGCCAGTGAGCTTCCCGTTCAG |
| SPRY1 | Forward | AAGCCATCAGAGGCAGCAAT |
| (for MeRIP-qPCR) | Reverse | TTTTCGGGTCTTGGTGCAGT |

**Table S3. The sequences of siRNAs used in this study.**

| Names | Sequence (5’-3’) | |
| --- | --- | --- |
| Si-NC | sense | UUCUCCGAACGUGUCACGUTT |
|  | antisense | ACGUGACACGUUCGGAGAATT |
| si-METTL3 #1 | sense | GCUGCACUUCAGACGAAUUAUTT |
|  | antisense | AUAAUUCGUCUGAAGUGCAGCTT |
| si-METTL3 #2 | sense | CGUCAGUAUCUUGGGCAAAUUTT |
|  | antisense | AAUUUGCCCAAGAUACUGACGTT |
| si-METTL3 #3 | sense | GCACCCGCAAGAUUGAGUUAUTT |
|  | antisense | AUAACUCAAUCUUGCGGGUGCTT |
| si-IGF2BP1 #1 | sense | GCAGUAUGUAGGCGCUAUCAUTT |
|  | antisense | AUGAUAGCGCCUACAUACUGCTT |
| si-IGF2BP1 #2 | sense | GAAACACCUGACUCCAAAGUUTT |
|  | antisense | AACUUUGGAGUCAGGUGUUUCTT |
| si-IGF2BP1 #3 | sense | CGACCAAGUCAUUGUUAAGAUTT |
|  | antisense | AUCUUAACAAUGACUUGGUCGTT |
| si-IGF2BP2 #1 | sense | CCUCUCGGGUAAAGUGGAAUUTT |
|  | antisense | AAUUCCACUUUACCCGAGAGGTT |
| si-IGF2BP2 #2 | sense | CCUUGCAGGAUUUGAGCAUUUTT |
|  | antisense | AAAUGCUCAAAUCCUGCAAGGTT |
| si-IGF2BP2 #3 | sense | CGGAUCUUUGGGAAACUGAAATT |
|  | antisense | UUUCAGUUUCCCAAAGAUCCGTT |
| si-IGF2BP3 #1 | sense | CCUUAGACAAACUGAAUGGAUTT |
|  | antisense | AUCCAUUCAGUUUGUCUAAGGTT |
| si-IGF2BP3 #2 | sense | CGCGGAGAAGUCCAUUACUAUTT |
|  | antisense | AUAGUAAUGGACUUCUCCGCGTT |
| si-IGF2BP3 #3 | sense | CCUACCCACAAUUUGAGCAAUTT |
|  | antisense | AUUGCUCAAAUUGUGGGUAGGTT |
| si-SPRY1 #1 | sense | UUGGUAUUAGAGGCAGAUUTT |
|  | antisense | AAUCUGCCUCUAAUACCAATT |
| si-SPRY1 #2 | sense | UGGACUUAGUUUGGUUUAUTT |
|  | antisense | AUAAACCAAACUAAGUCCATT |
| si-SPRY1 #3 | sense | UGCUUAAAUAAGCUAUAUATT |
|  | antisense | UAUAUAGCUUAUUUAAGCATT |
