# Supplementary material for: ADAP-METTL3 modulates the inflammatory responses of macrophages via m6A modification of Spry1
Source: Cell Death Dis. 2025 Oct 7;16(1):708. doi: 10.1038/s41419-025-08008-x (PMC12504520; doi:10.1038/s41419-025-08008-x)

Fig. 1A

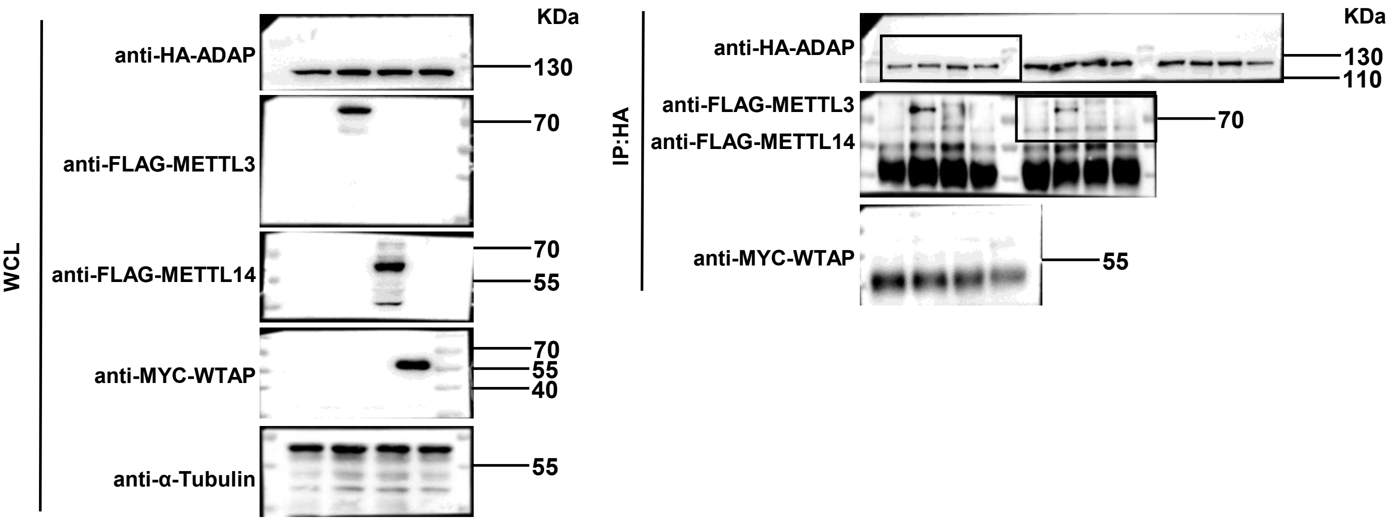

Fig. 1B

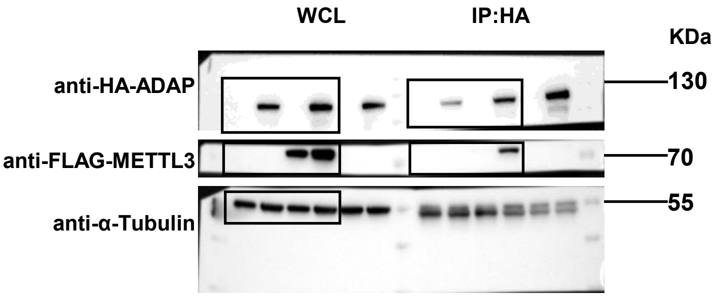

Fig. 1C

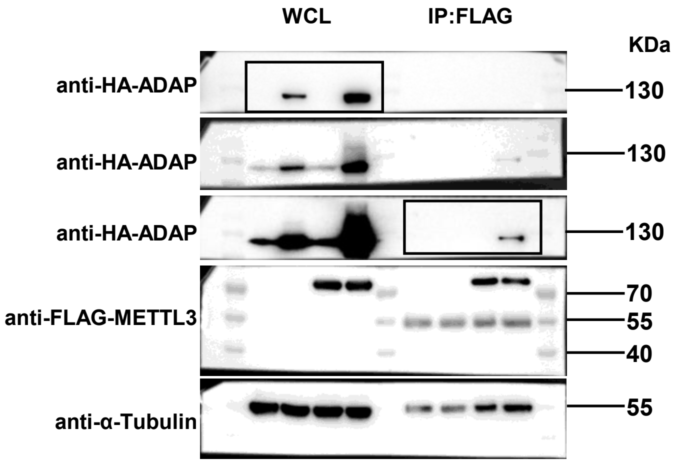

Fig. 1D

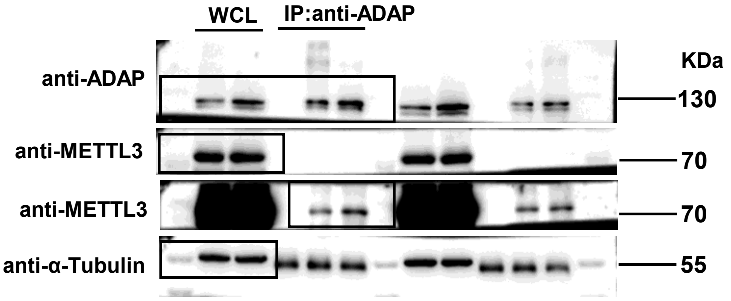

Fig. 1F

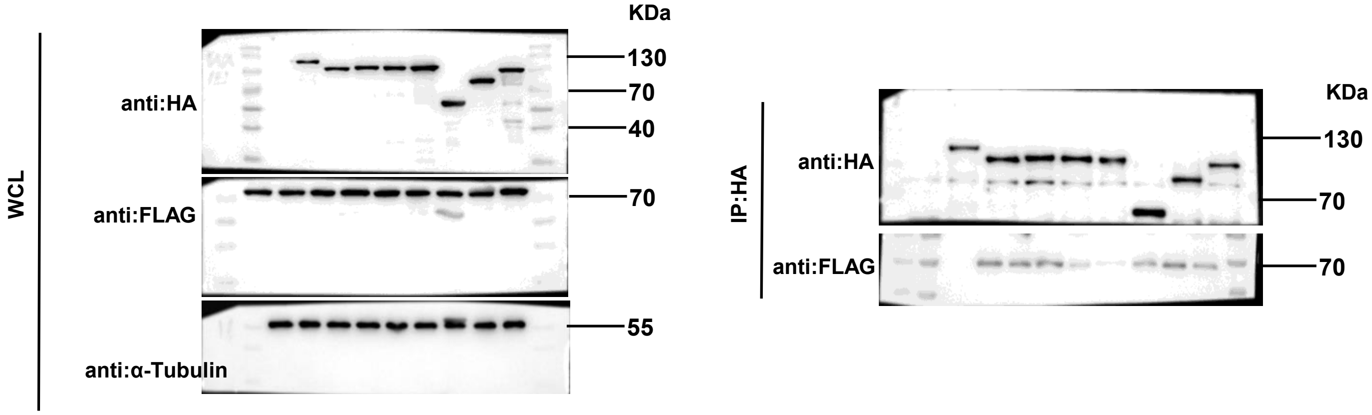

Fig. 1G

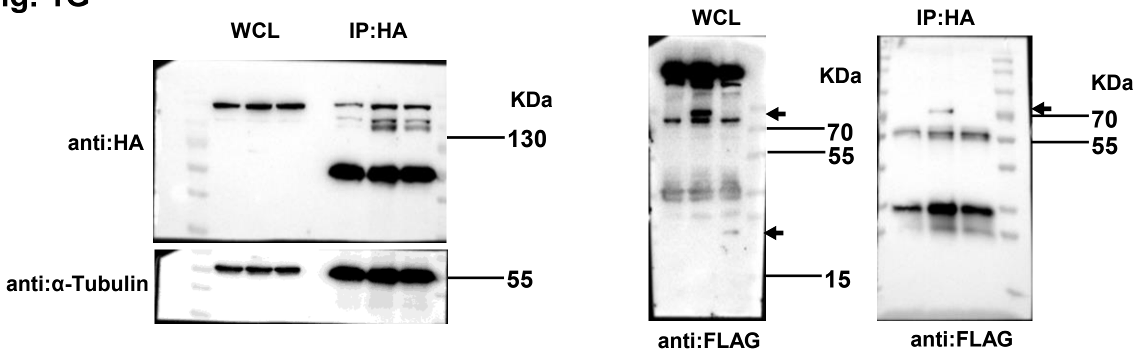

Fig. 5K

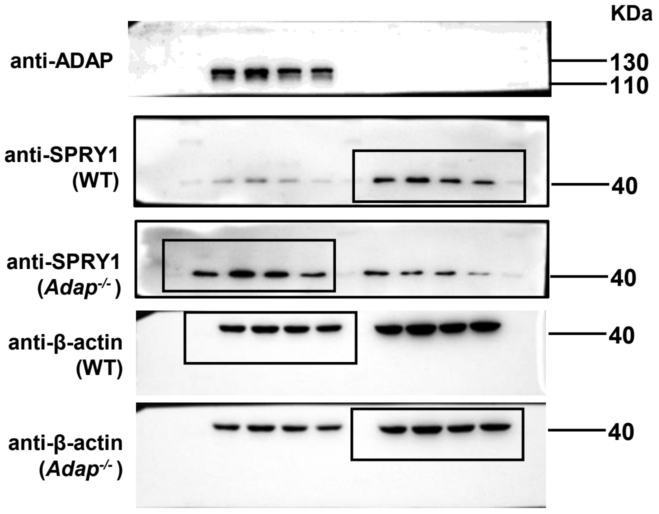

Fig. 5L

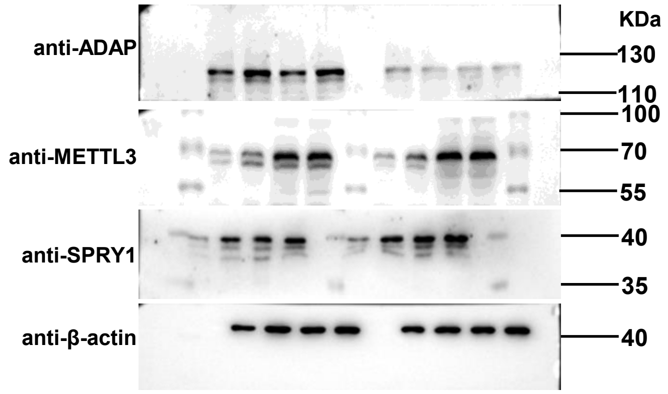

Fig. 6L

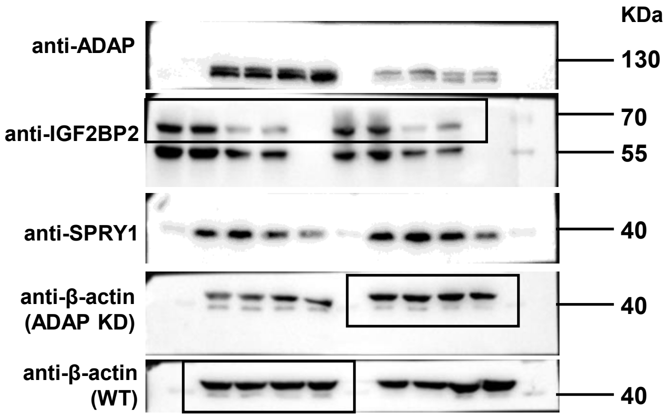

Fig. 6M

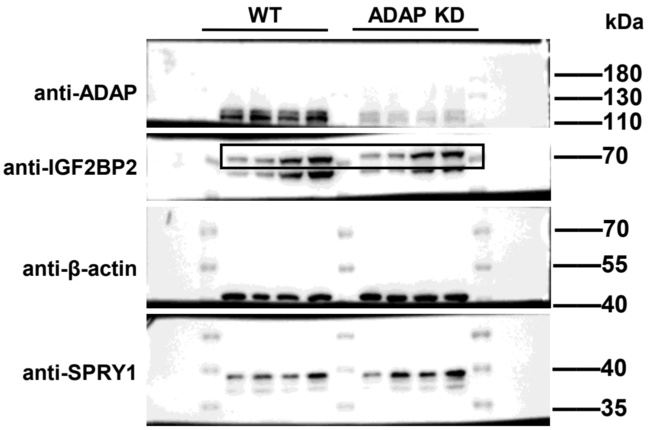

Fig. 7D

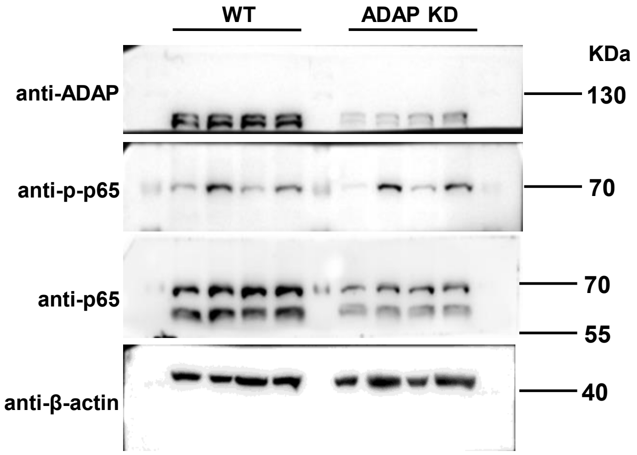

Fig. 7H

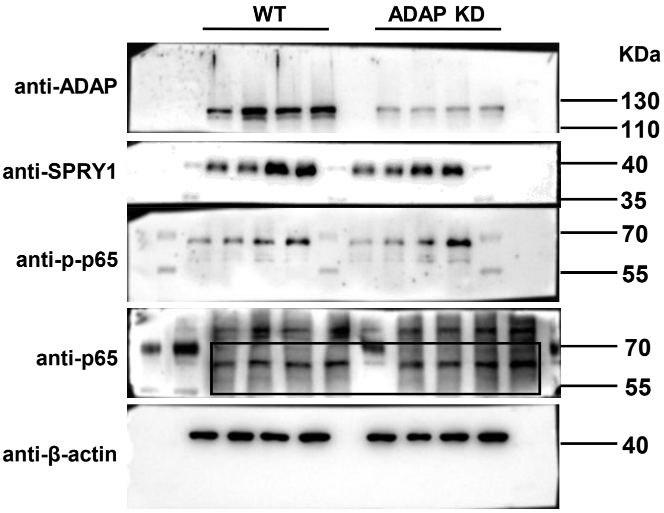

Fig. S1 P

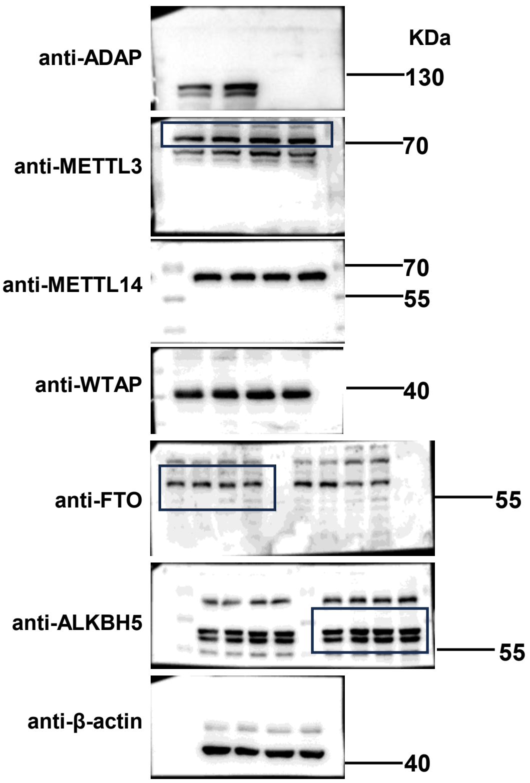

Fig. S2 B

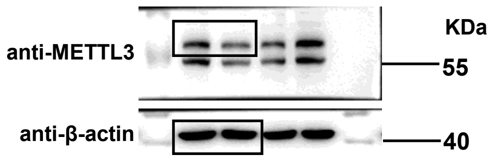

Fig. S2 C

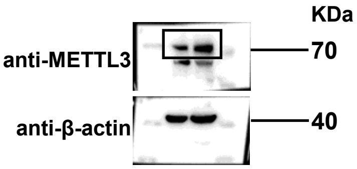

Fig. S2 D

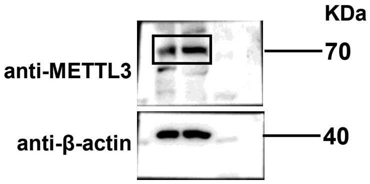

Fig. S2 H

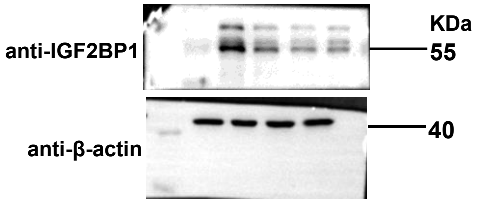

Fig. S2 I

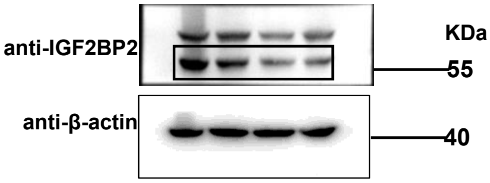

Fig. S2 J

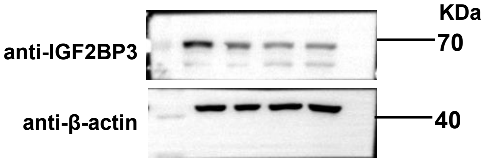

Fig. S2 K

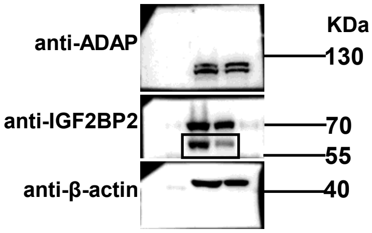

Fig. S2 L

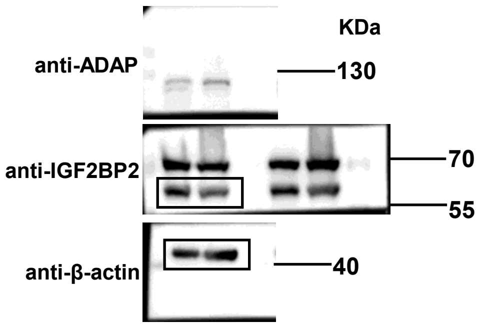

Fig. S2 M

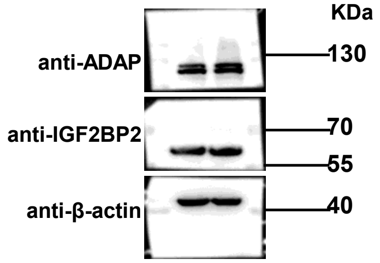

Fig. S2 N

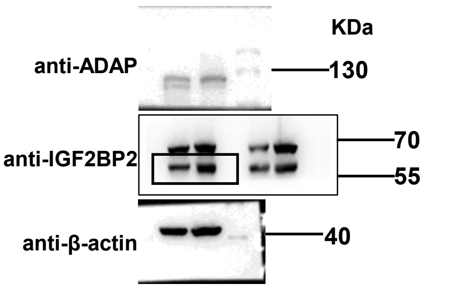

Fig. S2 P

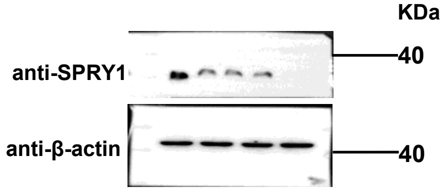

Fig. S2 Q

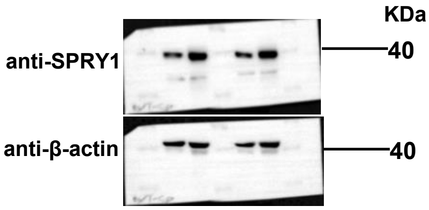

Fig. S2 R

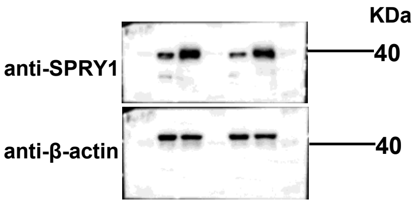

Fig. S3 E

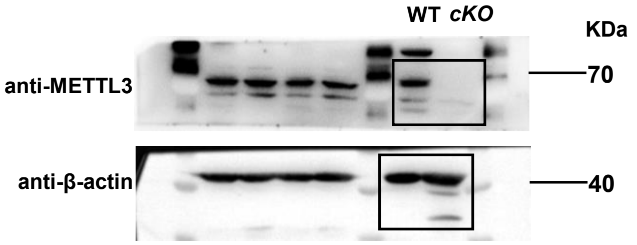

Fig. S3 F

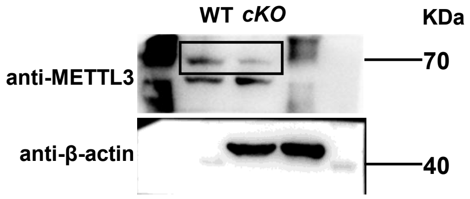

Fig. S3 G

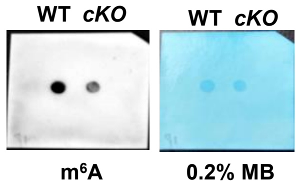

Fig. S3 H

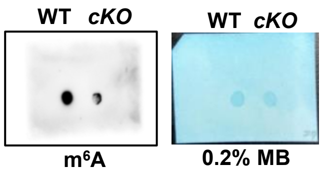

Fig. S3 J

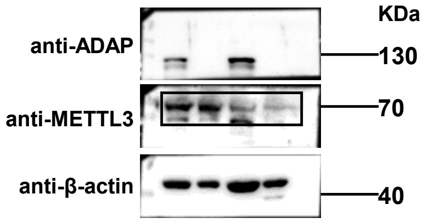

Supplement: Supplementary file 9 — Original western blots [file 41419_2025_8008_MOESM9_ESM.pdf]
